# The effect of rhamnolipids on fungal membrane models as described by their interactions with phospholipids and sterols: An *in silico* study

**DOI:** 10.3389/fchem.2023.1124129

**Published:** 2023-02-21

**Authors:** Nely Rodríguez-Moraga, Francisco Ramos-Martín, Sébastien Buchoux, Sonia Rippa, Nicola D’Amelio, Catherine Sarazin

**Affiliations:** ^1^ Unité de Génie Enzymatique et Cellulaire UMR 7025 CNRS, Université de Picardie Jules Verne, Amiens, France; ^2^ Unité de Génie Enzymatique et Cellulaire, CNRS UMR 7025, Sorbonne Universités, Université de Technologie de Compiègne, Compiègne, France

**Keywords:** rhamnolipids, molecular dynamics, coarse-grained, membranes, antifungal, biophysics, lipids, all-atom

## Abstract

**Introduction:** Rhamnolipids (RLs) are secondary metabolites naturally produced by bacteria of the genera *Pseudomonas* and Burkholderia with biosurfactant properties. A specific interest raised from their potential as biocontrol agents for crop culture protection in regard to direct antifungal and elicitor activities. As for other amphiphilic compounds, a direct interaction with membrane lipids has been suggested as the key feature for the perception and subsequent activity of RLs.

**Methods:** Molecular Dynamics (MD) simulations are used in this work to provide an atomistic description of their interactions with different membranous lipids and focusing on their antifungal properties.

**Results and discussion:** Our results suggest the insertion of RLs into the modelled bilayers just below the plane drawn by lipid phosphate groups, a placement that is effective in promoting significant membrane fluidification of the hydrophobic core. This localization is promoted by the formation of ionic bonds between the carboxylate group of RLs and the amino group of the phosphatidylethanolamine (PE) or phosphatidylserine (PS) headgroups. Moreover, RL acyl chains adhere to the ergosterol structure, forming a significantly higher number of van der Waals contact with respect to what is observed for phospholipid acyl chains. All these interactions might be essential for the membranotropic-driven biological actions of RLs.

## 1 Introduction

**FIGURE 1:**
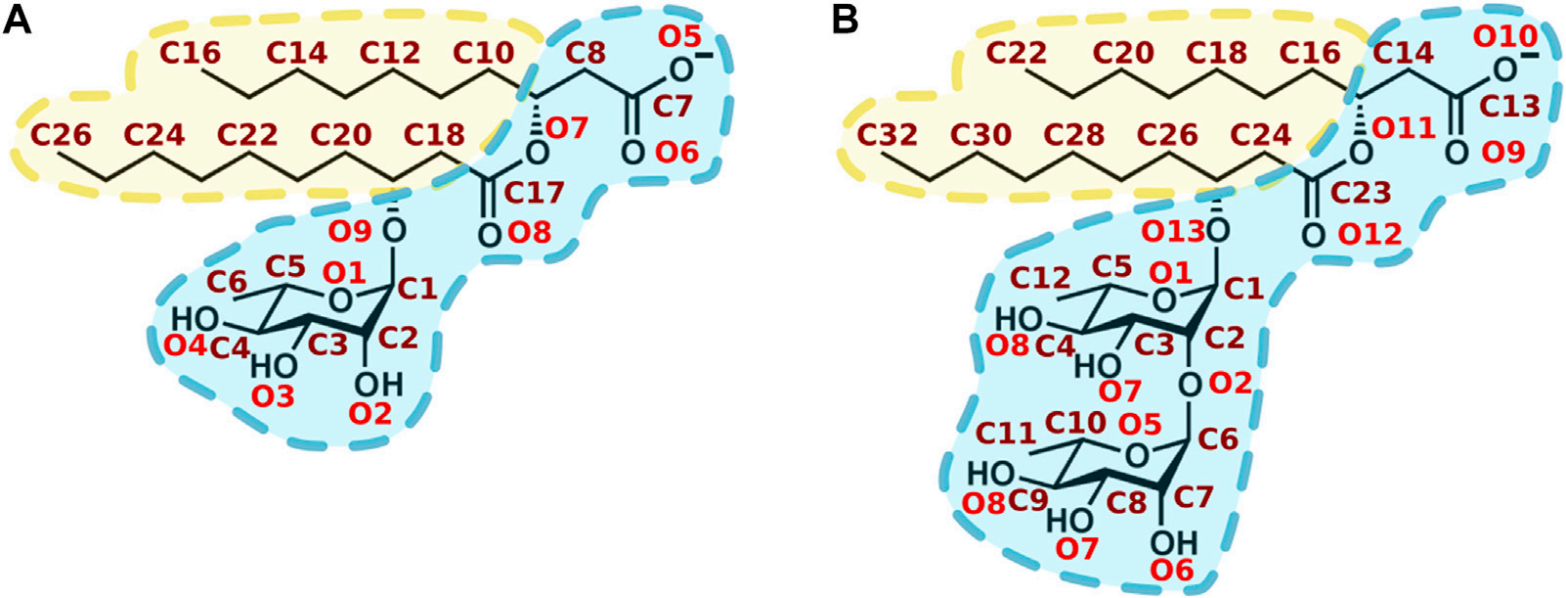
Chemical structure of main rhamnolipids produced by *Pseudomonas aeruginosa*. **(A)** α-L-rhamnopyranosyl-β-hydroxydecanoyl-β-hydroxydecanoate (mono-RL). **(B)** 2-O-α-L-rhamnopyranosyl-α-L-rhamnopyranosy-β-hydroxydecanoyl-β-hydroxydecanoate (di-RL). Oxygen and carbon atoms are respectively labelled in red and maroon. Hydrophilic and hydrophobic moieties are respectively shown in blue and in yellow.

Rhamnolipids (RLs) are amphiphilic glycolipid biosurfactants produced as secondary metabolites by several bacteria such as *Pseudomonas* and Burkholderia. RLs produced by *Pseudomonas aeruginosa* are the most studied, and contain mainly a mixture of mono- and di-rhamnose units (polar head) linked through a O-bond to one or two 3-hydroxy fatty acids (from 8 to 16 carbons). The most representative compounds are the α-L-rhamnopyranosyl-β-hydroxydecanoyl-β-hydroxy-decanoate (mono-RL) and 2-O-α-L-rhamnopyranosyl-α-L-rhamnopyranosyl-β-hydroxydecanoyl-β-hydroxydecanoate (di-RL) (Figure 1).

Aside from their use as detergents or in cosmetics (Randhawa et al., 2014; SantosDanyelle Khadydja et al., 2016), their interest stems from the fact that they display on the one hand antifungal properties, either with preventive and curative applications (Monnier et al., 2020) and, on the other hand, can trigger defence mechanisms in plants, making them ideal for sustainable crop-protection (Kumar and Amar Jyoti Das, 2018; Monnier et al., 2018; Crouzet et al., 2020). Although the role of receptors cannot be ruled out (Kutschera et al., 2019), their antifungal properties seem to be related to their direct interaction with the lipids from the target plasma membrane (Sha and Qin, 2016; Goswami et al., 2015; Abdel et al, 2010; Kim, Lee, and Hwang, 2000; Stanghellini and Miller, 1997; Crouzet et al., 2020; Robineau et al., 2020). Their membranotropic mode of action is less likely to induce development of pathogen resistances (Avis, 2007).

A direct interaction between the RL and the lipids from the plant plasma membranes has been proposed to explain their eliciting properties, in analogy with the mode of action of the lipopeptide surfactin which shares with RLs properties such as amphiphilic structure, small size and eliciting and antimicrobial activities (Henry et al., 2011; Ranf, 2017). This has been observed in *Arabidopsis thaliana* (Schellenberger et al., 2021) as well as in other Brassicaceae of agricultural interest or other plants commonly used in agriculture like grapevines, cherry-tomato fruit, or rapeseed (Varnier et al., 2009; Sanchez et al., 2012; Yan et al., 2016; Monnier et al., 2018).

The nature of membrane lipids can vary greatly across different organisms leading to tremendous structural diversity: as of 2022, more than 47,000 unique lipid structures have been reported in the LIPID MAPS Structure Database (LSMD) (Sud et al., 2006; Liebisch et al., 2020). Despite the great complexity of the plasma membrane composition, only three classes of lipids constitute the vast majority of eukaryotic membranes: glycerolipids (including phospholipids), sphingolipids, and sterols, offering the possibility to prepare simplified models for biophysical studies (Deleu et al., 2014; Ramos-Martín et al., 2022).

Focusing on the fungal kingdom, PC, PE, glycolipids, sphingolipids and ergosterol (ERGO) are the most abundant lipids although PS and phosphatidylinositol (PI) can also play important roles in some species and environmental conditions (Barran and Miller, 1976; Manocha, 1980; Weete, 1980; Lösel, 1990; Palma-Guerrero et al., 2010; Liu et al., 2015; Luo et al., 2015; Hasim et al., 2018; Khandelwal et al., 2018; Bassilana, Puerner, and Arkowitz, 2020; Perczyk et al., 2020; Santos et al., 2020; Ramos-Martín and D'Amelio, 2022). Cholesterol (CHOL) and ergosterol are found mainly in animal and fungal plasma membranes (Thevissen et al., 2003; Perczyk et al., 2020), respectively, whereas β-sitosterol (SITO) and stigmasterol (STIGM) are typical of plant plasma membranes (Valitova, Sulkarnayeva, and Minibayeva, 2016).

In the present work, we focus on glycerophospholipids and sterols as these systems have been widely used to understand the action of RLs and other antifungal agents. On the other hand, the contribution of other lipid types such as glycolipids or sphingolipids must not be underestimated and would deserve a dedicated study. For example, sphingolipids have been shown to be key in the formation of highly rigid gel domains, are involved in important physiological processes and may contribute to the action of several antifungal agents (F. C. Santos et al., 2020; Aresta-Branco et al., 2011; Vecer et al., 2014; Körner and Fröhlich, 2022; Olsen and Færgeman, 2017; Leal and Suarez. 2022).

By using phospholipid-based membrane mimetic models, it has been possible to show that RLs have the capability to influence membrane fluidity, a property highly dependent on phospholipid acyl chain length, saturation degree (Hazel, 1995; Ernst, Ejsing, and Antonny, 2016; Sezgin et al., 2017; Fonseca et al., 2019; Mondal et al., 2020) and the concentration of sterols (Hartmann, 1998; Piironen et al., 2000; Vance, 2000; Volkman, 2003). In particular, di-RLs are able to alter the lipid dynamics and lower the temperature of the gel-to-fluid phase transition (Ortiz et al., 2006). Other studies showed that at a higher RL-to-lipid ratio (larger than one), di-RLs induce leakage of POPC (1-palmitoyl-2-oleoyl-glycero-3-phosphocholine) vesicles (Sánchez et al., 2010) thus proving evidence of the destructive influence that RLs can have on biological membranes. Our previous results suggested a differentiating role of ergosterol in the fluidity of glycerophospholipid/sterols models (Monnier et al., 2019).

In this work, we highlight key intermolecular interactions accounting for the effect of RLs on biological membranes with the aim to provide an insight into the mechanism of action underpinning their antifungal activity. By examining a large variety of phospholipids with different saturation degrees and sterols, we show how the formation of intermolecular H-bonds, salt bridges and van der Waals contacts all cooperate to define the exact location of RLs inside different membrane bilayers. In all cases, such placement determines a significant fluidification of the membrane, likely leading to important consequences on the target organism. As described in the “Results and discussion” section, our study is able to explain many phenomena previously observed with other techniques, such as membrane fluidification and the affinity of RLs for PE and sterols present in fungal organisms. By dissecting our initial complex systems into its simple components we have provided an analytical description of key interactions which can form the basis for further development of antifungal agents with desired properties.

## 2 Materials and methods

All simulations were run using GROMACS software (Abraham et al., 2015). The structural properties of membrane thickness, membrane area and area per lipid were calculated by FATSLiM software (Buchoux, 2017). VMD [158] was used for visualisation. Graphs and images were created with Xmgrace, Inkscape (Project, 2020), GNUplot (Janert, 2016) and PyMol (DeLano, 2002).

### 2.1 Coarse grained simulations

#### 2.1.1 System setup

Coarse Grained (CG) simulations were carried out using the MARTINI 2 force field (Marrink, de Vries, and Mark, 2004; Marrink et al., 2007). Membranes were built with the insane.py script available in the Martini web page (Wassenaar et al., 2015). The script was adapted to add RLs in the membranes.

All CG simulations were performed with a time step of 14 fs under periodic boundary conditions applied in all directions for a total simulation time length of 15 µs. For long range interactions, the reaction-field option was used. For the short-range electrostatic and van der Waals interactions, the Verlet cutoffs (Páll and Hess, 2013) were fixed to 1.1 nm. All systems were studied at 299 K with the V-rescale thermostat (Bussi, Donadio, and Parrinello, 2007) in combination with a time constant of 1.0 ps. The Parrinello-Rahman barostat (Parrinello and Rahman, 1981) was used to maintain a mean average pressure of one bar in a semi-isotropic manner with a compressibility of 3.0 × 10^–4^ bar^-1^ and a coupling constant of 12 ps.

All simulations were minimised using the steepest descent algorithm and then equilibrated for a total time of about 4 ns during which the time step was gradually increased (1 fs, 5 fs, 10 fs, and 14 fs) while decreasing the constraints on polar head movements (from 200, 100, 50, 20 to 10 kJ mol^-1^ nm^-2^).

#### 2.1.2 RLs and lipid CG topologies

**FIGURE 2:**
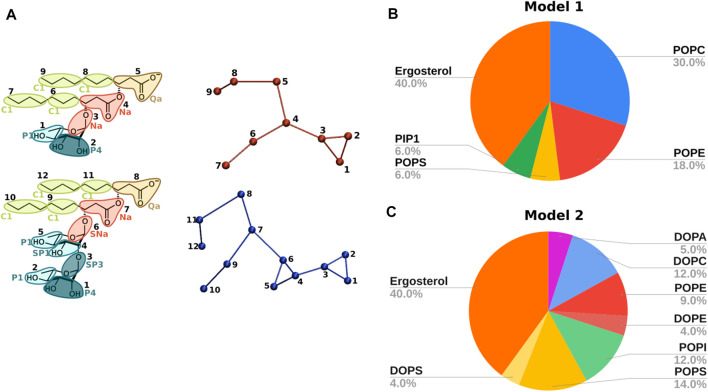
**(A)** CG topologies for mono-RL (top) and di-RL (bottom) with Martini bead assignments. Mapping colours refer to different bead types and the different tonalities to the polarity degree. Apolar, charged, intermediate and polar beads are respectively represented with green, brown, red and turquoise. **(B)** Phospholipid membrane model reproducing the composition of Fusarium oxysporum: POPC, POPE, POPS, PIP1 and ergosterol in molar ratios 30/18/6/6/40. **(C)** Phospholipid membrane model reproducing the composition of *Saccharomyces cerevisiae*: DOPA, DOPC, POPE, DOPE, POPI, POPS, DOPS and ergosterol in molar ratios 5/12/9/4/12/14/4/40.

Lipidic moieties were described by common Martini parameters. RLs CG topologies (not available in the Martini FF) were built starting from those of monogalactosyldiacylglycerol (MGDG) and digalactosyldiacylglycerol (DGDG) in their anionic form (López et al., 2013; van Eerden et al., 2015). Out of many different models available, differing in the mapping, Martini bead types, rhamnose/s bead bonds, dihedral angles as well as constraint and/or exclusion definitions, we selected the one shown in Figure 2 on the basis of the comparison of results obtained by all-atom (AA) and CG simulations. In particular, a RL (mono- or di-RL) was simulated in both water (10 ns) and POPC membrane (128 total lipids, 500 ns) using both C36 (2 fs time steps) and Martini 2 (20 fs time step) FFs.

First, CG distributions of bonds, angles and dihedral angles were compared to the atomistic ones (for the comparison we used the centre of mass of atoms corresponding to each CG bead). The choice of the best model required several tests until a good reproduction of CG bonds, angles and dihedral angles was achieved (see Supplementary Figures S1, S2 for the optimised mono- and di-RLs topologies, shown in Supplementary Tables S1, S2).

To ensure that the chosen CG topologies could also reproduce membrane properties, we monitored both area per lipid and membrane thickness of (a) RL-containing POPC membrane as well as for (b) a more complex model containing POPC/POPG/ergosterol (53/23/25) and 1 RL/25 lipids (40/60, mono- and di-RL) (see Supplementary Figure S3). Both models allowed us to compare the results with previous works from our group (Monnier et al., 2019). These latter models were run with 256 lipids, 4 mono-RLs and 6 di-RLs for 500 ns (AA) and with 864 lipids, 9 mono-RLs and 27 di-RLs for 10 µs (CG).

For model (a), membrane properties were nicely reproduced with stable trajectories (20 fs time step). Good results were also achieved for model (b) (see Supplementary Figure S3) when reducing the time step (14 fs), as previously reported for other complex glycolipids (López et al., 2013).

The final parameters used for mono- and di-RLs topologies are listed in Supplementary Tables S1, S2. They correspond to those of the model which gave the best results in terms of system stability, bond, angle and dihedral angle distributions (Supplementary Figure S1), and structural membrane properties (Supplementary Figure S3). Mappings and Martini bead assignments are shown in Figure 2A.

#### 2.1.3 Simulated lipid systems

In the present work, the following models have been studied by means of CG simulations (Figures 2B, C) (described further in the text in the Results and Discussion Section 3.1).

- Model 1: POPC (1-palmitoyl-2-oleoyl-glycero-3-phosphocholine), POPE(1-palmitoyl-2-oleoyl-sn-glycero-3-phosphoethanolamine), POPS (1-palmitoyl-2-oleoyl-sn-glycero-3-phospho-L-serine), PIP1 (phosphatidylinositol phosphate) and ergosterol (30/18/6/6/40).
- Model 2: DOPA (1,2-dioleoyl-sn-glycero-3-phosphate), DOPC (1,2-dioleoyl-sn-glycero-3-phosphocholine), POPE, DOPE (1,2-dioleoyl-sn-glycero-3-phosphoethanolamine), POPI (1-palmitoyl-2-oleoyl-sn-glycero-3-phosphoinositol), POPS, DOPS (1,2-dioleoyl-sn-glycero-3-phospho-L-serine) and ergosterol (5/12/9/4/12/14/4/40).

#### 2.1.4 Analysis

CG order parameters were calculated by the python script do-order-gmx5. py available on the MARTINI website (Ingolfsson, 2022) using the equation:

$$P_{2}=\frac{1}{2}\left(3\;\cos²\;<\Theta >-1\right)$$

P_2_ considers the angle θ formed by the bonds of two consecutive CG beads and the normal of the membrane (axis z). Values of 1, -0.5 and 0 correspond to a perfect alignment, anti-alignment and random orientation with respect to the membrane normal, respectively.

Data were analysed by GROMACS tools (gmx command): density, rdf (for radial distribution function), and (for lateral diffusion). More details about how the calculations are performed are described in detail in GROMACS manual (Lindahl and Spoel. 2022).

Ergosterol flip-flops were calculated using LiPyphilic toolkit (Smith and Lorenz, 2021). A successful flip-flop event was defined as a translocation from one leaflet to another followed by permanence in the destination leaflet for at least 10 ns, as previously done for other works (Gu, Baoukina, and Tieleman, 2019).

### 2.2 All atom simulations

#### 2.2.1 System setup

AA MD simulations were carried out with Charmm36 (C36) force field (Huang and MacKerell, 2013), using a time integration step of 2 fs under periodic boundary conditions applied in all directions and with an overall time length of 500 ns. The Particle Mesh Ewald (PME) method (Essmann et al., 1995) was used for long-range interactions. For short-range electrostatic and van der Waals interactions, the Verlet cutoff radii (Páll and Hess, 2013) was set at 1.2 nm. The systems were studied at 299 K. The Nosé-Hoover temperature coupling (Hoover, 1985; Nosé, 2002) was used with a time constant of 0.5 ps. The average pressure was maintained at one bar by the Parrinello-Rahman barostat (Parrinello and Rahman, 1981) in a semi-isotropic fashion (pressure in the XY plane independent of the pressure along Z) with a compressibility of 4.5 × 10^−5^ bar^-1^ and a coupling constant of 5 ps. All bonds were constrained with the LINCS algorithm (Hess et al., 1997). Water molecules were described by the TIP3P model (Jorgensen et al., 1983). All simulations were minimised using the steepest descent algorithm. Equilibrations were carried out using the 6-step protocol provided by CHARMM-GUI for 375 ps.

A water layer of 45 Å thickness was added above and below the lipid bilayer of 128 lipids (64 per leaflet) which resulted in about 12,000 water molecules being incorporated, the exact number depending on the nature of the membrane. Systems were neutralised with K⁺ counterions. Binary systems phospholipid/sterol and the ternary model POPE/POPI/ERGO were mixed in ratio 70/30 and 35/35/30, respectively. The whole process (minimization, equilibration and production run) was repeated once.

“Ligand Reader and Modeller” from CHARMM-GUI was used to generate CHARMM-compatible topologies and parameters files for the anionic RLs form. Penalties reported by CgenFF were between the recommended values for well parametrized molecules (Kim et al., 2017). RLs were later incorporated to the membrane using the usual CHARMM-GUI workflow (Jo et al., 2009) and placed either over the upper leaflet at a non-interacting distance (>10 Å) or placed inside the bilayer at the beginning of the simulation.

#### 2.2.2 Simulated lipid systems

Multiple lipid systems have been evaluated during the work.

- Single lipid models containing POPC, POPE, POPS, POPI or DLiPE (1,2-dilinoleoyl-sn-glycero-3-phosphoethanolamine).
- Binary models containing mixtures of phospholipids with cholesterol (CHOL), ergosterol (ERGO), sitosterol (SITO) or stigmasterol (STIGM) (in 70/30 M ratios).
- Ternary systems containing POPE/POPI/ERGO (35/35/30 M ratios).

#### 2.2.3 Analysis

Data were analysed by GROMACS tools (gmx command): density (for electron density profiles), potential (for dipole potential calculations), order (for order parameter calculations), rdf (for radial distribution function), and (for lateral diffusion). More details about how the calculations are performed are described in detail in GROMACS manual (Lindahl and Spoel, 2022). Polar and apolar contacts were analysed using radial distribution function by an in-house developed script.

## 3 Results and discussion

### 3.1 Coarse grained molecular dynamics of fungal membranes

Molecular dynamics simulations of biological membranes require quite extensive sampling due to the fact that some processes, such as RL aggregation, or flip-flop lipid interconversions, take place from sub-milliseconds to even minute (Ingolfsson et al., 2015). For this reason, we decided to study the action of RLs in fungal membrane models by CG MD simulations, which allowed us to have a more extensive picture of their effect on membrane properties in a longer time scale and even to detect sterol and phospholipid flip-flops (Supplementary Table S3).

To take into account the complexity of fungal membranes, we chose two models (Figures 2B, C) inspired by the literature (Monje-Galvan and Klauda, 2015; Ermakova and Zuev, 2017).

The first model features five phospholipid components and reproduces the composition of Fusarium oxysporum, an opportunistic pathogen able to infect important agricultural products such as citrus fruit (Snowdon, 1990), potatoes, onions, garlic and maize among many others (Pitt and Hocking, 2009). Such a model, composed of POPC, POPE, POPS, PIP1 and ergosterol in ratios 30/18/6/6/40 (model 1, Figure 2B), also allowed us to compare and validate our results with previous studies (Ermakova and Zuev, 2017), before studying the interaction with RLs.

As a second model, we decided to simulate the closely related organism *Saccharomyces cerevisiae* which contains an even wider variety of headgroups: DOPA, DOPC, POPE, DOPE, POPI, POPS, DOPS and ergosterol in ratio 5/12/9/4/12/14/4/40 (model 2, Figure 2C) (Monje-Galvan and Klauda, 2015). In comparison with model 1, model 2 contains a larger amount of unsaturated acyl chains, as commonly seen in many fungal organisms as Botrytis cinerea (Griffiths et al., 2003).

#### 3.1.1 Rhamnolipids decrease membrane thickness and fluidify fungal-like membranes

The effect of rhamnolipids was investigated by introducing a mixture of mono and di-RLs in ratio 40/60 (1 RL for 25 lipids) to the model membranes (Monnier et al., 2019; 2020; Botcazon et al., 2022).

**FIGURE 3:**
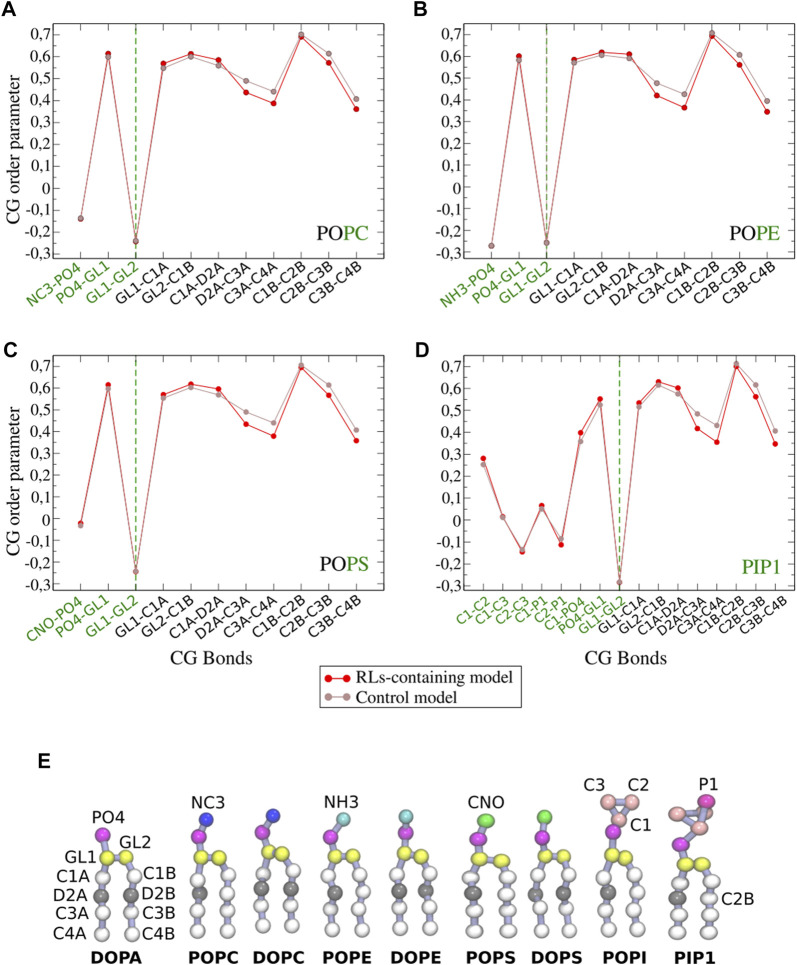
CG order parameters of each phospholipid **(A–D)** in model 1 (POPC/POPE/POPS/PIP1/ergosterol 30/18/6/6/40) with and without RLs (shown in red and brown, respectively). Bonds implied in modelled polar heads are shown in green (see Figure 2D for CG bead names). **(E)** Structure of phospholipids in coarse grained simulations.

- Model 1: Our simulation of model 1 closely reproduced previous data (Ermakova and Zuev, 2017) in terms of both structural (membrane thickness, area per lipid) and dynamical properties (lipid diffusion and CG order parameters) (Supplementary Table S3). Such mono:di-RL and lipid:RL ratios reproduce previous studies where a fluidification effect was observed by 2H-NMR in some sterol-containing membrane models (Monnier et al., 2019) (see Figures 3A–D). For the model 1, which contains only one unsaturation per lipid, we observe a slight decrease in membrane thickness, consistent with an increase in membrane fluidity (Lemkul, 2019; Moradi, Nowroozi, and Shahlaei, 2019) as monitored by CG order parameters (Figure 3). Lipid diffusion appears unaltered (Supplementary Table S3).
- Model 2: In the presence of RLs, membrane thickness and area per lipid do not change significantly for model 2 (Supplementary Table S3). Fluidification effect appears less evident than for model 1 (Supplementary Figure S4), probably due to a weaker action on RLs with this system containing many more unsaturations.

An increased fluidity is in very good agreement with experimental observations reported to date, that also point to the modification of phase behaviour due to the RLs action (Sánchez et al., 2006; Oliva et al., 2020). Indeed, this was observed before for RLs in DMPC or DMPS models for which the presence of RLs broadens and shifts the transition to lower temperatures. Similar effect was detected for more complex models composed of DPPC/DPPG/DOPC/DOPG/cholesterol in molar ratio 45/5/20/5/25 (Herzog et al., 2020). Other studies using Atomic force microscopy (AFM) showed that the insertion of the RLs in the latter anionic model caused a reversal of the normal proportion between the liquid ordered (68.5%) and liquid disordered (31.5%) phases (Herzog et al., 2020). Finally, this is in coherence with fluorescence microscopy studies that revealed that the presence of mono-RLs in giant unilamellar vesicles (GUVs) causes the disappearance of pre-existing liquid ordered phase (Herzog et al., 2020).

### 3.2 All atom molecular dynamics simulations

Coarse-grained molecular dynamics simulations have the great advantage to allow the study of large and complex systems over a relatively long time interval. However, key interatomic interactions such as the formation of hydrogen bonds or other interatomic contacts cannot be evaluated. Highlighting such interactions can be extremely informative to understand the molecular causes of macroscopic effects (e.g., membrane fluidification).

Moreover, the parametrization of different sterol types in AA C36 force field for finer structural description allow us to evaluate more lipid types as those present in plant membranes, likely involved in RLs eliciting effect (Bauer et al., 2006; Varnier et al., 2009). For these reasons we decided to run all atom MD simulations of simplified membrane models.

#### 3.2.1 Rhamnolipids are able to penetrate all types of tested mimetic systems

We took advantage of the higher resolution of AA models to get insight on how RLs are able to get internalised into their target membranes. To this end, placing rhamnolipids (mono-RLs and di-RLs) well outside the surface, we simulated different systems for a long time (up to 3 µs).

RLs penetrate PE-containing membranes much more often and easily than PC- ones, including those containing sterols. This finding might be explained by the significant difference in the steric hindrance of the choline moiety of PC headgroup with respect to the amino group of PE. Moreover, we observe a quicker penetration when sterols are added to the membrane models as discussed further in the text.

#### 3.2.2 Rhamnolipids are localised just beneath the plane formed by membrane phosphate groups

As we observe some degree of internalisation in all PE-based systems, independently of the internalisation time, we decided to focus on systems where all RLs are internalised from the start.

To take into account the role of intrinsic fluidity of the membrane and its possible influence on the action of RLs (see Section 3.2.5), each phospholipid was studied with two different types of acyl chains, one with only one insaturation (oleic acyl chain) and one with 6 insaturations (3 insaturations per acyl chain). The effect of sterols was also taken into account as all simulations were run in the absence and in the presence of ergosterol. Besides the commonly studied cholesterol, β-sitosterol and stigmasterol were studied too, allowing to ascertain the differences each sterol could produce in the RLs action (Supplementary Figure S5). The effect of RLs was studied with both mono and di-RLs.

**FIGURE 4:**
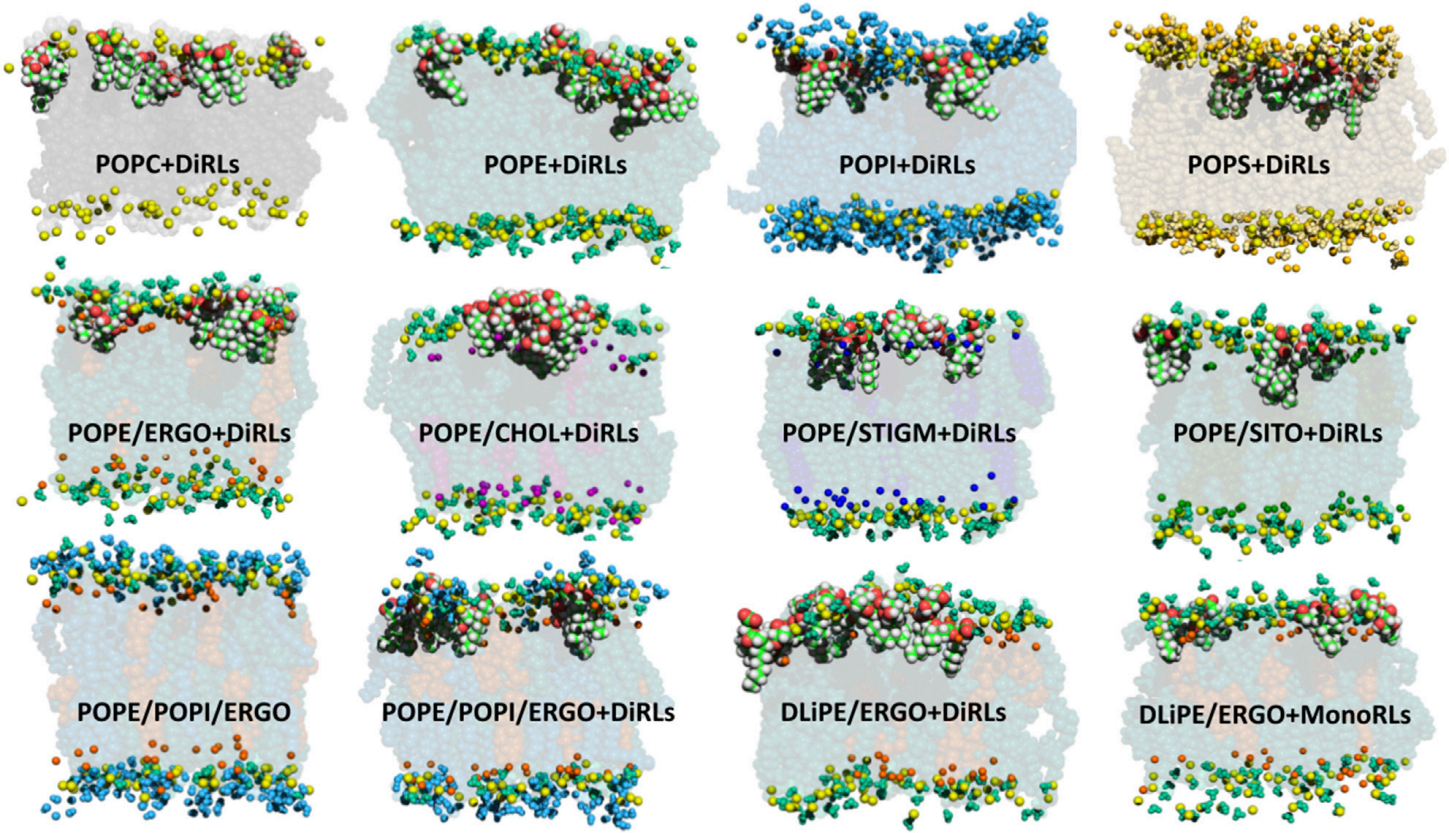
MD snapshots showing RLs internalised in several membranes with variable phospholipid compositions. Colour code: phosphorus atom (yellow); POPC body (black) and choline group (light grey); POPE body (dark green), headgroup (turquoise) and amine of the headgroup (light green); POPS body (brown), headgroup (gold), amine of the headgroup (light yellow) and carboxyl of the headgroup (orange); POPI body (blue), headgroup (light blue) and hydroxyls of the headgroup (cyan); CHOL body (purple) and hydroxyl (light purple); ERGO body (dark orange) and hydroxyl (light orange); SITO body (deep-dark green) and hydroxyl (pea-green); STIGM body (electric blue) and hydroxyl (light electric blue). For clarity, only functional groups of headgroups are represented (spheres) in the upper leaflet. RLs are shown as van der Waal spheres (C in green, H in white, O in red). Binary models contain phospholipid:sterol mixtures at a molar ratio of 70/30.

Figure 4 shows snapshots of RLs interacting with a variety of membranes. In most cases, RLs are found well inserted into the bilayers with the rhamnose head in close proximity and just beneath the phosphate groups. RLs acyl chains flank those of phospholipids, especially in the presence of PE or PS headgroups, both playing an important role in several fungal organisms (Cassilly and Reynolds, 2018).

#### 3.2.3 H-bonds and a salt bridges stabilise the localization of rhamnolipids within membranes

**FIGURE 5:**
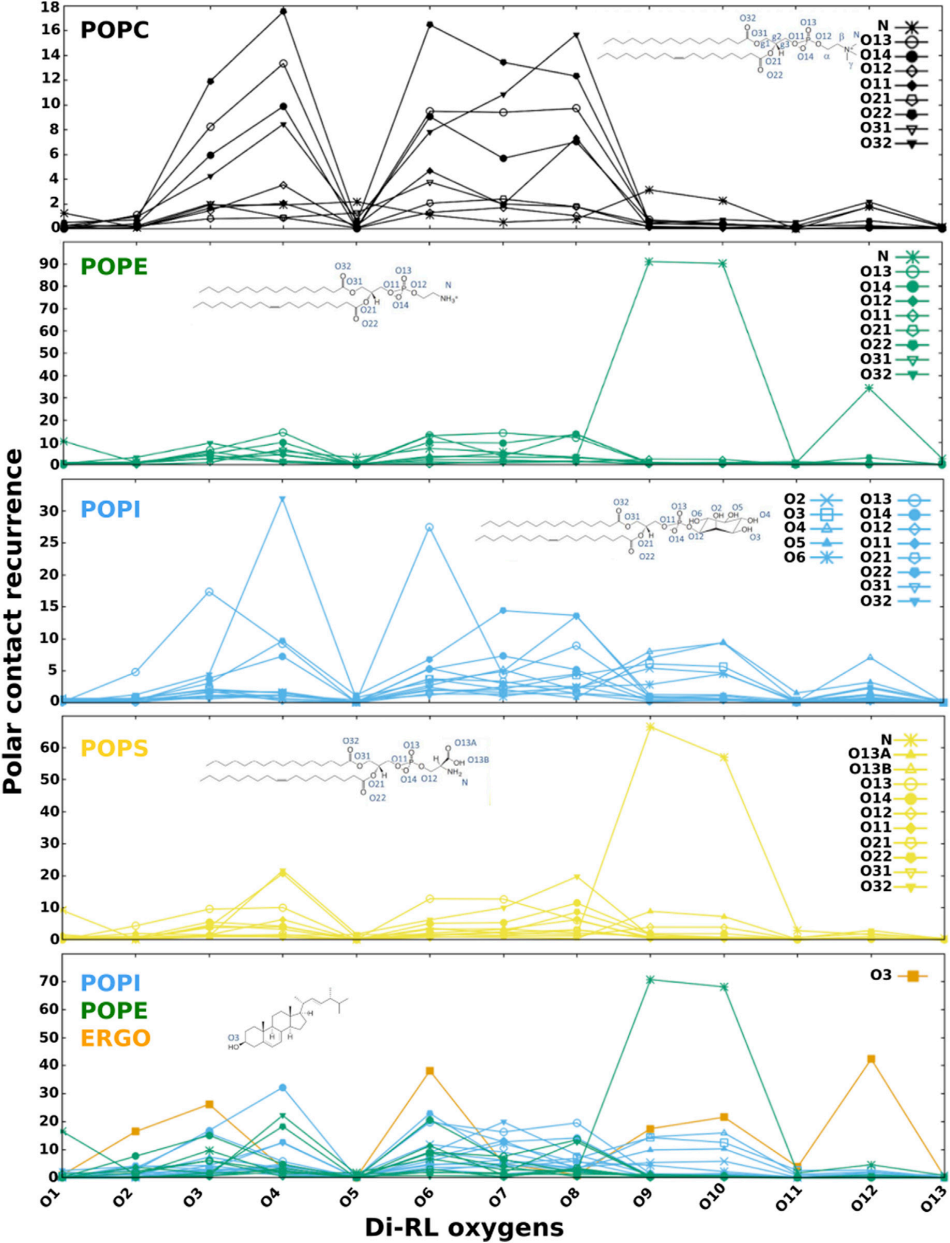
Recurrence of close contacts between polar atoms of di-RLs and those of phospholipids, indicating the formation of H-bonds or salt bridges. Membrane models: POPC; POPE; POPI; POPS; a simplified model of fungal membrane composed of POPE, POPI and ERGO (35/35/30).

**FIGURE 6:**
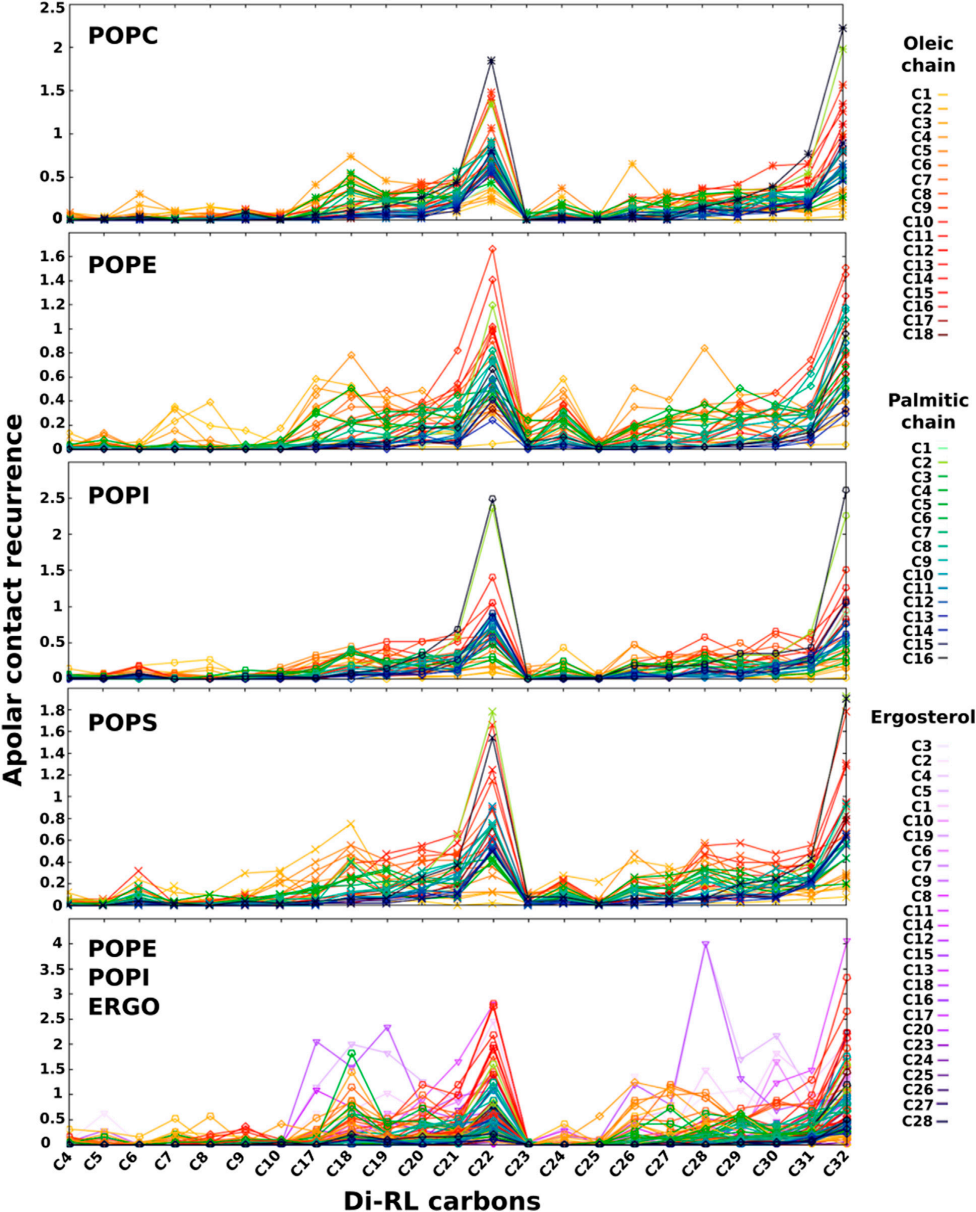
Recurrence of close contacts between apolar atoms of di-RLs and those of phospholipids and ergosterol, indicating the formation of van der Waals contacts. Membrane models: POPC; POPE; POPI; POPS; a simplified model of fungal membrane composed of POPE, POPI and ERGO (35/35/30). Atoms coloured from light green to dark blue refer to oleic acyl chain while those coloured from yellow to dark red refer to palmitic acyl chain. Colour tonality reflects the distance from the headgroup. Carbons are numbered in order from the carbonyl group for palmitic and oleic chains. Ergosterol carbon numbering is depicted in Supplementary Figures S5.

**FIGURE 7:**
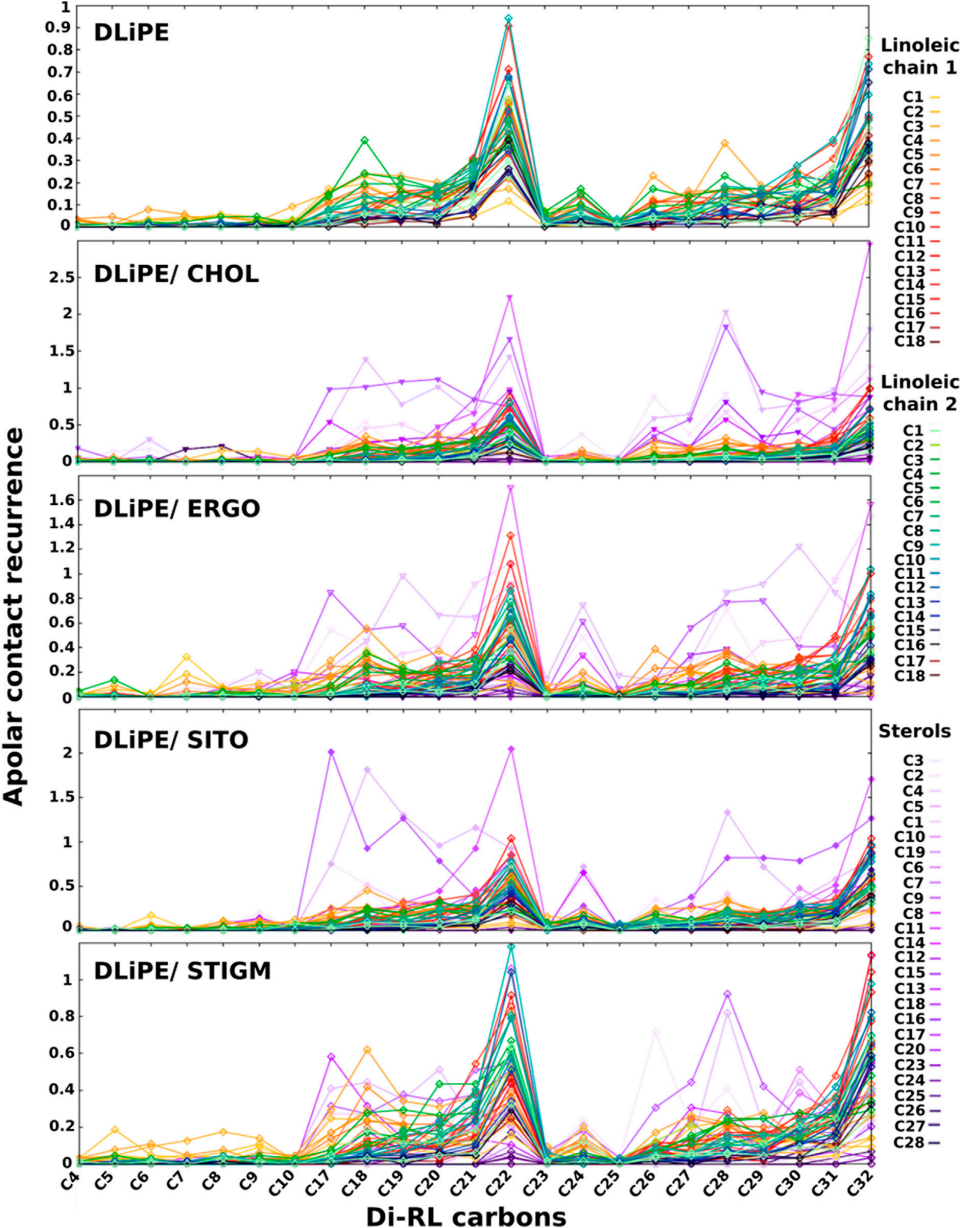
Recurrence of close contacts between apolar atoms of di-RLs and those of phospholipids, indicating the formation of van der Waals contacts. DLiPE; DLiPE/CHOL; DLiPE/ERGO; DLiPE/SITO; DLiPE/STIGM (70/30 M ratios). Colour tonality reflects the distance from the headgroup. Carbons are numbered in order from the carbonyl group for linoleic chains. Sterols’ carbon numbering is depicted in Supplementary Figures S5.

In order to get insight into the interactions favouring the described localization, we analysed polar and apolar contacts (Figures 5–7, Supplementary Figures S6–S10) in the simulations with RLs initially placed inside (to mimic final RLs distribution in the membrane after their full internalisation). MD simulations have previously reported the formation of hydrogen bonds between water and the carbonyl group of DMPS (Oliva et al., 2020). Figure 5 clearly show that RLs only rarely form polar contacts with PC, accounting for RLs selectivity, that would be important to avoid toxicity effects on cells exposing more PC in their outer leaflets (Phoenix et al., 2015; Cassilly and Reynolds, 2018; Ramos-Martín and D'Amelio, 2022). On the contrary, a frequent and dominant interaction (Figure 5) is the formation of a salt bridge between the amino group of PE (or PS) and the carboxylate of RL (atoms O5 and O6 in mono-RLs and O9 and O10 in di-RLs, Figure 1).

PI behaves similarly to PC but in this case the rhamnose oxygen atoms form sporadic H-bonds with its phosphate or the hydroxyl groups of the inositol. Dominant interactions are more evident when we analyse a mixture of phospholipids representing a simplified fungal membrane (PE/PI/ERGO, Figure 5). In this case, the salt bridge with PE largely dominates over all other polar contacts. It is worth noticing that the localization of RLs in the membrane is ideal for the formation of H-bonds between hydroxyl groups of the rhamnose and that of ergosterol.

We believe that the formation of the salt bridge between RLs and the amino group of PE (or PS) plays a role in keeping RL at the observed position. When we analyse the apolar contacts (Figure 6) we find that the acyl chain of RLs make significant van der Waals interactions with those of phospholipids and such contacts become more frequent as we move away from the rhamnose. Interestingly, in the case of PC and PI, RL chains are capable of making contacts with atoms of phospholipid chains localised close to the core of the bilayer (in Figure 6 atom colours become darker as we move away from the headgroups), indicating that they are not well anchored to the surface and confirming the role of the salt bridge with the PE (or PS) headgroup. In all cases, contacts with unsaturated chains (oleoyl) seem slightly favoured over those with saturated ones (palmitic).

Our observations on RL localization in the bilayer are coherent with previous FTIR studies showing that the presence of di-RLs affects the C=O bonds of the polar heads of a DPPC model and, to a lesser extent, the bonds of the hydrophobic chain (Sánchez et al., 2009). As for the importance of the salt bridge, isothermal calorimetric titration (ITC) studies have shown that, compared to liposomes formed only by POPC, di-RLs have a higher affinity for those composed of POPC/POPE (1/1) but less affinity for liposomes containing POPC/cholesterol (1/1) (Aranda et al., 2007).

#### 3.2.4 Rhamnolipids association with sterols

FTIR combined with molecular modelling of an implicit membrane (hypermatrix) showed that the presence of sterols tends to increase the interaction of RLs with membrane lipids (Nasir et al., 2017).

These data seem to indicate that sterols may play an important role in the interaction of RLs with biomembranes. We therefore investigated apolar contacts between RLs and several sterols (Figure 7, Supplementary Figures S9, S10). Our data show that, while RL acyl chains interact frequently with phospholipids with their terminal ends, they seem to adhere completely to sterols when they are present. Such interactions (in violet in Figures 7, Supplementary Figure S9) are important and tend to dominate over all other apolar contacts both with the more rigid (containing palmitoyl and oleoyl acyl chains) and more fluid membranes (containing dilinoleyl-acyl chains, shown in Figure 7). It should be noted that similar effects are found also in POPC/CHOL systems (Supplementary Figure S10), when forcing RLs to be inserted in the membrane at the start of our simulations. Conversely, while free floating RLs spontaneously insert in PE-containing bilayers, they are unable to integrate mammalian models made of POPC/CHOL at least in the time range explored (2 μs), thus providing a possible explanation for the selectivity.

**FIGURE 8:**
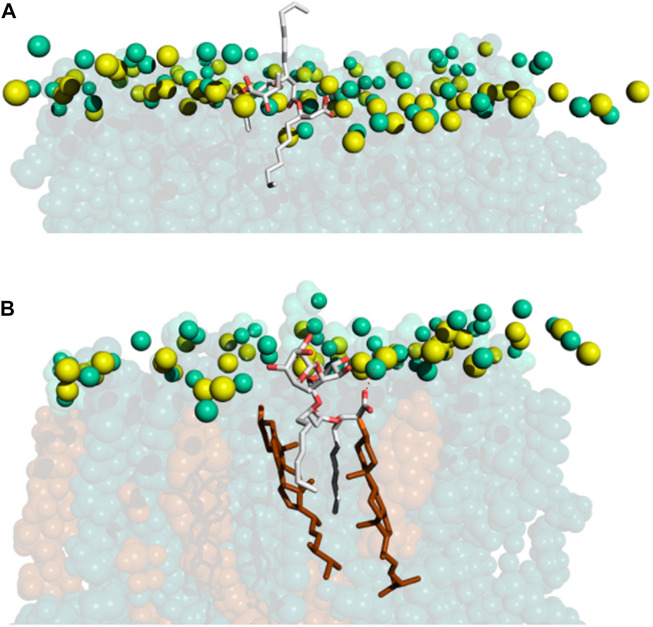
MD snapshots showing how **(A)** RLs get inserted in POPE/ERGO (70/30) model membranes and how, **(B)** once inside, they recruit ergosterol, forming microdomains. RLs are represented in sticks (carbon in grey and oxygen in red). For membrane colouring refer to the caption of Figure 4. Electrostatic interactions are shown as red dotted lines.

Indeed, our data are consistent with previous literature and provide a molecular picture of the interaction (Figure 8). RLs approach the membrane surface by its polar head. In the case of POPE the driving force is the electrostatic interaction between its carboxylate and the amino group of POPE. Although RLs tend to aggregate before they get inserted, they usually penetrate the membrane one-by-one. After reaching the surface, RLs insert one acyl chain in the hydrophobic part of the bilayer (Figure 8A). In the case of POPE, the bridge with the amino group is maintained as the molecule further descends into the bilayer, facilitating the entrance of the second RL chain. Once inserted, the acyl chains of RLs recruit two (or more) sterol molecules (Figure 8B). The remarkably similar length of RL acyl chains and ring structure of sterols maximises van der Waals interactions. In this localization the rhamnose rings can form H-bonds with polar atoms of phospholipid head groups while the salt bridge between the RL carboxylate and the amino group persists. Occasionally the carboxylate can also form H-bonds with the hydroxyl of sterols, especially in phospholipids lacking the amino groups. Rarely it can also make intramolecular H-bonds with its rhamnose and descend deeper in the bilayer.

#### 3.2.5 Rhamnolipids alterations on structural membrane features

Our coarse grained simulations involving complex fungal membrane models (Figures 2B, C) have highlighted a fluidification of biomembranes caused by the addition of RLs (Figure 3), an effect observed also in other models by Laurdan-based fluorescence spectroscopy (Herzog et al., 2020), 2H-NMR and MD simulations (Monnier et al., 2019).

In general, mono-RLs and di-RLs effects are similar in trends and slightly more intense for di-RLs. Therefore, for simplicity we will focus on DiRLs.

**FIGURE 9:**
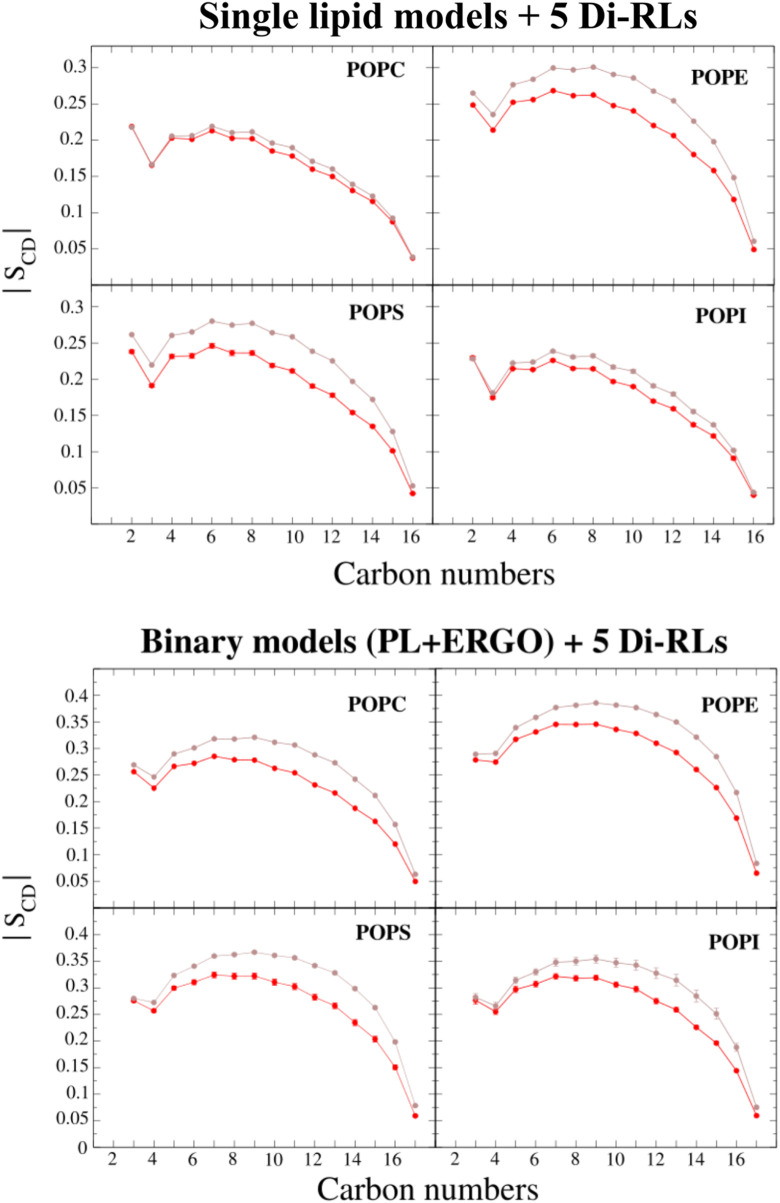
The deuterium order parameters compared with the carbon position of the sn-1 acyl chain of single-PL membrane models (POPC, POPE, POPS and POPI) and binary membrane models (POPC/ERGO, POPE/ERGO, POPS/ERGO and POPI/ERGO, 70/30 M ratios) with and without di-RLs (shown in red and brown, respectively). The error bars represent the standard deviation of the last averaged 250 ns (smaller than the symbol size in some cases).

Fluidification can be monitored by changes in the order parameter of phospholipid acyl chains (Figure 9 and Supplementary Figure S11). As it was observed in our CG simulations, also in AA simulations we observe a larger fluidification for membranes with only one unsaturation (POP-phospholipids) rather than those bearing 6 unsaturations (DLi-phospholipids).

Analysing electron density profiles, we can evaluate lipid interdigitation processes or an increase in the amount of water molecules near the polar head groups due to loose packaging. The POPE model displays stronger changes than POPC model (Supplementary Figure S13), showing an increase in the density in the central part of the membrane due to lipid interdigitation and a reduction of the density in the headgroups zone, probably due to the intercalation of additional water molecules. The presence of sterols significantly reduces the effect in mixed POPE models (especially sitosterol and stigmasterol). Similar phenomena are observed for other phospholipids often found in fungi such PS and PI (Supplementary Figure S14). On the other hand, no big changes are observed between single and mixed POPC-based models (Supplementary Figure S15).

The dipole potential ΨD originates from the alignment of dipolar parts of the lipids and water molecules (Wang, 2012; Clarke, 2001; Pearlstein, Dickson, and Hornak, 2017; Brockman, 1994; Ramos-Martín and D’Amelio, 2022). The magnitude of the dipole potential is dependent on the structure of the lipid, the degree of unsaturation and the nature of the linkage between headgroup and hydrocarbon chain (ester or ether). Intercalation of dipolar molecules such as some antimicrobial peptides can greatly modify the potential, leading to significant membrane damage (Dreyer et al., 2013; Pearlstein, Dickson, and Hornak, 2017). Changes in the dipole potential are in fact able to affect the translocation rates of ions across the bilayer and the partition and translocation of macromolecules. The gating kinetics of voltage-gated ion channels can also be altered by the modulation of ΨD. This mechanism of action is employed by some antifungal agents (Zakharova et al., 2019) due to the fact that ΨD is important for fungal survival (Zhao and Tombola, 2021), as in eukaryotes for the regulation of cardiac or neuronal processes (Brockman, 1994; Pearlstein, Dickson, and Hornak, 2017).

Given the importance of ΨD for fungal survival, we examined changes in dipole potential in single-lipid models. The most important reduction is observed for pure POPE (Supplementary Figure S16). Interestingly, the presence of sterols reduces the effect of RLs in all different types of membranes examined (Supplementary Figures S16–S19).

Regarding other parameters, such as the area per lipid, we can observe a slight increment due to RLs incorporation to the membranes, specially in POP-models (Supplementary Figure S20). Membrane thickness (Supplementary Figure S21) slightly decreases in all the membrane models, coherently with the reduction of the hydrophobic thickness that can be monitored by electron density profiles (Supplementary Figure S13).

## 4 Conclusion

In this work we have provided an atomistic description of the interaction of RLs with multiple membranes and especially fungal models in view of their antifungal activity. Our simulations show that RLs penetrate more easily and form more interactions with membranes rich in PE, usually exposed in the outer leaflet of many fungi, than PC whose headgroups are exposed in mammal cells. In particular we have shown how RLs get internalised in membrane models and localise beneath the plane formed by phosphate groups, thanks to important interactions involving their carboxylate moiety and amino groups of PE and PS phospholipids. In this position, RL acyl chains can form vdW contacts with those of PLs and quite stable adducts with ergosterol, thus significantly modifying membrane fluidity. We would like to explore the importance of such interaction in future works to better understand the role of sterols in the plant-defence eliciting property of PLs. Indeed, recent works have reported the key role of sphingolipids in the eliciting action of RLs in plants like *A. thaliana* (Schellenberger et al., 2021). Not only sphingolipids facilitate the entrance of the RLs in plant membranes (rich on PC in the outer leaflet (Cordelier et al., 2021)) but also promote the formation of microdomains (Yu et al., 2020a) that would stimulate proteins responsible for the induction of immune response in plants (Cordelier et al., 2021). Even more, sphingolipids promote the incorporation of sterols to these domains (Yu et al., 2020a), with important consequences for immune response and other important physiological roles involving protein-protein or protein-lipid interactions (Yu et al., 2020a; Cordelier et al., 2021). The micro RL/sterols domains that we describe in this work could contribute to the overall picture.

## Data Availability

The original contributions presented in the study are included in the article/Supplementary Material, further inquiries can be directed to the corresponding authors.

## Abbreviations

CHOL, cholesterol; DGDG, digalactosyldiacylglycerol; di-RL, 2-O-α-L-rhamnopyranosyl-α-L-rhamnopyranosyl-β-hydroxydecanoyl-β-hydroxydecanoate; mono-RL, α-L-rhamnopyranosyl-β-hydroxydecanoyl-β-hydroxy-decanoate; DLiPE,1,2-dilinoleoyl-sn-glycero-3-phosphoethanolamine;DMPC,1,2-dimyristoyl-sn-glycero-3-phosphocholine; DOPA, 1,2-dioleoyl-sn-glycero-3-phosphate; DOPC,1,2-dioleoyl-sn-glycero-3-phosphocholine;DOPE, 1,2-dioleoyl-sn-glycero-3-phosphoethanolamine; DOPS1,2-dioleoyl-sn-glycero-3-phospho-L-serine; DPPC, 1,2-dipalmitoyl-sn-glycero-3-phosphocholine; DSPC, 1,2-distearoyl-sn-glycero-3-phosphocholine; DLiPE1,2-dilinoleoyl-sn-glycero-3-phosphoethanolamine)DEPE:1,2-dielaidoyl-sn-glycero-3-phosphoethanolamine;DMPS:1,2-dimyristoyl-sn-glycero-3-phospho-L-serine; ERGO, ergosterol; LINCS, LINear Constraint Solver; MGDG, monogalactosyldiacylglycerol; NPT, isothermal-isobaric ensemble; PBC, Periodic Boundary Conditions; PE, phosphatidylethanolamine;PG,phosphatidylglycérol; PI, phosphatidylinositol; PL, Phospholipid; PME,particlemeshEwald;POPC:1-palmitoyl-2-oleoyl-glycero-3-phosphocholine;POPE,1-palmitoyl-2-oleoyl-sn-glycero-3-phosphoethanolamine; POPI,1-palmitoyl-2-oleoyl-sn-glycero-3-phosphoinositol; POPS, 1-palmitoyl-2-oleoyl-sn-glycero-3-phospho-L-serine; PS, phosphatidylserine; RL, rhamnolipid; RLs, Rhamnolipids; SITO, sitosterol; SM, sphingomyelin; STIGM, stigmasterol.

## References

[B1] Abdel M. Ahmad M. Lépine F. Déziel E. (2010). Rhamnolipids: Diversity of structures, microbial origins and roles. Appl. Microbiol. Biotechnol. 86 (5), 1323–1336. 10.1007/s00253-010-2498-2 20336292 PMC2854365

[B2] Abraham M. J. Murtola T. Schulz R. Páll S. Smith J. C. Hess B. (2015). Gromacs: High performance molecular simulations through multi-level parallelism from laptops to supercomputers. SoftwareX. 10.1016/j.softx.2015.06.001

[B3] Aranda F. J. Espuny M. J. Marqués A. Teruel J. A. Manresa A. Ortiz A. (2007). Thermodynamics of the interaction of a dirhamnolipid biosurfactant secreted by Pseudomonas aeruginosa with phospholipid membranes. Langmuir ACS J. Surfaces Colloids 23 (5), 2700–2705. 10.1021/la061464z 17243729

[B4] Aresta-Branco F. André M. C. Susana Marinho H. Cyrne L. Antunes F. Rodrigode Almeida F. M. de Almeida R. F. (2011). Gel domains in the plasma membrane of Saccharomyces cerevisiae: Highly ordered, ergosterol-free, and sphingolipid-enriched lipid rafts. J. Biol. Chem. 286 (7), 5043–5054. 10.1074/jbc.m110.154435 21127065 PMC3037616

[B5] Avis T. J. (2007). Antifungal compounds that target fungal membranes: Applications in plant disease control. Rev. Can. Phytopathol. 29 (4), 323–329. 10.1080/07060660709507478

[B6] Barran L. R. Miller R. W. Roche I. d. l. (1976). Temperature-induced alterations in phospholipids of Fusarium oxysporum F. Sp. Lycopersici. Can. J. Microbiol. 22 (4), 557–562. 10.1139/m76-083 1260546

[B7] Bassilana M. Puerner C. Arkowitz R. A. (2020). External signal-mediated polarized growth in fungi. Curr. Opin. Cell Biol. 62, 150–158. 10.1016/j.ceb.2019.11.001 31875532

[B8] Bauer J. Brandenburg K. Ulrich Z. Rademann J. (2006). Chemical synthesis of a glycolipid library by a solid-phase strategy allows elucidation of the structural specificity of immunostimulation by rhamnolipids. Chemistry 12 (27), 7116–7124. 10.1002/chem.200600482 16915594

[B9] Botcazon C. Bergia T. Lecouturier D. Dupuis C. Rochex A. Acket S. (2022). Rhamnolipids and fengycins, very promising amphiphilic antifungal compounds from bacteria secretomes, act on *Sclerotiniaceae* fungi through different mechanisms. Front. Microbiol. 13, 977633. 10.3389/fmicb.2022.977633 36246282 PMC9557291

[B10] Brockman H. (1994). Dipole potential of lipid membranes. Chem. Phys. Lipids 73 (1-2), 57–79. 10.1016/0009-3084(94)90174-0 8001185

[B11] Buchoux S. (2017). FATSLiM: A fast and robust software to analyze MD simulations of membranes. Bioinformatics 33 (1), 133–134. 10.1093/bioinformatics/btw563 27578804

[B12] Bussi G. Donadio D. Parrinello M. (2007). Canonical sampling through velocity rescaling. J. Chem. Phys. 126 (1), 014101. 10.1063/1.2408420 17212484

[B13] Cassilly C. D. Reynolds T. B. (2018). PS, it’s complicated: The roles of phosphatidylserine and phosphatidylethanolamine in the pathogenesis of Candida albicans and other microbial pathogens. J. Fungi (Basel, Switz. 4 (1), 28. 10.3390/jof4010028 PMC5872331 29461490

[B14] Clarke R. J. (2001). The dipole potential of phospholipid membranes and methods for its detection. Adv. Colloid Interface Sci. 89-90, 263–281. 10.1016/s0001-8686(00)00061-0 11215797

[B15] Cordelier S. Crouzet J. Gilliard G. Dorey S. Deleu M. Dhondt-Cordelier S. (2021). Deciphering the role of plant plasma membrane lipids in response to invasion patterns: How Biology and biophysics could help? J. Exp. Bot. November 73, 2765–2784. 10.1093/jxb/erab517 35560208

[B16] Crouzet J. Arguelles-Arias A. Dhondt-Cordelier S. Cordelier S. Pršić J. Hoff G. (2020). Biosurfactants in plant protection against diseases: Rhamnolipids and lipopeptides case study. Front. Bioeng. Biotechnol. 8, 1014. 10.3389/fbioe.2020.01014 33015005 PMC7505919

[B18] DeLano W. L. (2002). Pymol: An open-source molecular graphics tool. CCP4 Newsl. Protein Crystallogr. 40 (1), 82–92.

[B19] Deleu M. Crowet J-M Nasir M. N. Lins L. (2014). Complementary biophysical tools to investigate lipid specificity in the interaction between bioactive molecules and the plasma membrane: A review. Biochimica Biophysica Acta 1838 (12), 3171–3190. 10.1016/j.bbamem.2014.08.023 25175476

[B20] Dreyer J. Zhang C. Ippoliti E. Carloni P. (2013). Role of the membrane dipole potential for proton transport in gramicidin A embedded in a DMPC bilayer. J. Chem. Theory Comput. 9 (8), 3826–3831. 10.1021/ct400374n 26584128

[B22] Ermakova E. Zuev Y. (2017). Effect of ergosterol on the fungal membrane properties. All-atom and coarse-grained molecular dynamics study. Chem. Phys. Lipids 209, 45–53. 10.1016/j.chemphyslip.2017.11.006 29122611

[B23] Ernst R. Ejsing C. S. Bruno A. (2016). Homeoviscous adaptation and the regulation of membrane lipids. J. Mol. Biol. 428, 4776–4791. 10.1016/j.jmb.2016.08.013 27534816

[B24] Essmann U. Perera L. Berkowitz M. L. Darden T. Lee H. Pedersen L. G. (1995). A smooth particle mesh Ewald method. J. Chem. Phys. 103, 8577–8593. 10.1063/1.470117

[B25] Fonseca F Pénicaud C Elizabeth Tymczyszyn E. Gómez-Zavaglia A. Passot S. (2019). Factors influencing the membrane fluidity and the impact on production of lactic acid bacteria starters. Appl. Microbiol. Biotechnol. 103 (17), 6867–6883. 10.1007/s00253-019-10002-1 31300854

[B26] Goswami D Narayan Borah S Lahkar J Suresh D. (2015). Antifungal properties of rhamnolipid produced by Pseudomonas aeruginosa DS9 against colletotrichum falcatum. J. Basic Microbiol. 55 (11), 1265–1274. 10.1002/jobm.201500220 26173581

[B27] Griffiths R. G. Jane Dancer O’Neill E Harwood J L. (2003). Lipid composition of Botrytis cinerea and inhibition of its radiolabelling by the fungicide iprodione. New Phytologist 160 (1), 199–207. 10.1046/j.1469-8137.2003.00848.x 33873546

[B28] Gu R-X Baoukina S Peter Tieleman D. (2019). Cholesterol flip-flop in heterogeneous membranes. J. Chem. Theory Comput. 15 (3), 2064–2070. 10.1021/acs.jctc.8b00933 30633868

[B29] Hartmann M. (1998). Plant sterols and the membrane environment. Trends Plant Sci. 3 (5), 170–175. 10.1016/s1360-1385(98)01233-3

[B30] Hasim S. Vaughn E. N. Donohoe D. Gordon D. M. Pfiffner S. Reynolds T. B. (2018). Influence of phosphatidylserine and phosphatidylethanolamine on farnesol tolerance in Candida albicans. Yeast 35 (4), 343–351. 10.1002/yea.3297 29143357 PMC5893404

[B31] Hazel J. R. (1995). Thermal adaptation in biological membranes: Is homeoviscous adaptation the explanation? Annu. Rev. Physiology 57, 19–42. 10.1146/annurev.ph.57.030195.000315 7778864

[B32] Henry G Deleu M Jourdan E Thonart P Ongena M (2011). The bacterial lipopeptide surfactin targets the lipid fraction of the plant plasma membrane to trigger immune-related defence responses. Cell. Microbiol. 13 (11), 1824–1837. 10.1111/j.1462-5822.2011.01664.x 21838773

[B33] Herzog M. Tiso T. Blank L. M. Winter R. (2020). Interaction of rhamnolipids with model biomembranes of varying complexity. Biochimica Biophysica Acta, Biomembr. 1862 (11), 183431. 10.1016/j.bbamem.2020.183431 32750318

[B34] Hess B. Bekker H. HermanBerendsen J. C. Fraaije J G. E. (1997). Lincs: A linear constraint solver for molecular simulations. J. Comput. Chem. 18. 10.1002/(sici)1096-987x(199709)18:12<1463:aid-jcc4>3.0.co;2-h

[B35] Hoover W. G. (1985). Canonical dynamics: Equilibrium phase-space distributions. Phys. Rev. A General Phys. 31 (3), 1695–1697. 10.1103/physreva.31.1695 9895674

[B36] Huang J. MacKerell A. D. Jr (2013). CHARMM36 all-atom additive protein force field: Validation based on comparison to NMR data. J. Comput. Chem. 34 (25), 2135–2145. 10.1002/jcc.23354 23832629 PMC3800559

[B37] Ingolfsson H. I. (2022) Do-order. Available at: http://www.cgmartini.nl/index.php/downloads/tools/229-do-order (Accessed March 29, 2022).

[B38] Ingolfsson H I. Tieleman P Marrink S (2015). Lipid organization of the plasma membrane. Biophysical J. 108, 358a. 10.1016/j.bpj.2014.11.1962 25229711

[B39] Janert P K. (2016). Gnuplot in action: Understanding data with graphs. Manhattan, New York City: Simon and Schuster.

[B40] Jo S Lim J B. Klauda Jy B. Wonpil Im (2009). CHARMM-GUI membrane builder for mixed bilayers and its application to yeast membranes. Biophysical J. 97 (1), 41a–58a. 10.1016/j.bpj.2008.12.109 PMC2711372 19580743

[B41] Jorgensen W L. Chandrasekhar J Jeffry D. Madura R W. I Klein M L. (1983). Comparison of simple potential functions for simulating liquid water. J. Chem. Phys. 79, 926–935. 10.1063/1.445869

[B42] Khandelwal N. K. Sarkar P. Gaur N. A. Chattopadhyay A Prasad R (2018). Phosphatidylserine decarboxylase governs plasma membrane fluidity and impacts drug susceptibilities of Candida albicans cells. Biochimica Biophysica Acta, Biomembr. 1860 (11), 2308–2319. 10.1016/j.bbamem.2018.05.016 29856993

[B43] Kim B. S. JungLee Y. Hwang B. K. (2000). *In vivo* control and*in vitro* antifungal activity of rhamnolipid B, a glycolipid antibiotic, againstPhytophthora capsici andColletotrichum orbiculare. Pest Manag. Sci. 56, 1029–1035. 10.1002/1526-4998(200012)56:12<1029:aid-ps238>3.0.co;2-q

[B44] Kim S. Lee J. Jo S. CharlesBrooks L. 3rd Sun H. Wonpil I. (2017). CHARMM-GUI ligand reader and modeler for CHARMM force field generation of small molecules. J. Comput. Chem. 38 (21), 1879–1886. 10.1002/jcc.24829 28497616 PMC5488718

[B45] Körner C Fröhlich F (2022). Compartmentation and functions of sphingolipids. Curr. Opin. Cell Biol. 74, 104–111. 10.1016/j.ceb.2022.01.006 35228099

[B46] Kumar R Amar Jyoti Das (2018). Application of rhamnolipids in agriculture and food industry.” in rhamnolipid biosurfactant, 97–109. Singapore: Springer.

[B47] Kutschera A. Dawid C. Gisch N. Schmid C. Raasch L. Gerster T (2019). Bacterial medium-chain 3-hydroxy fatty acid metabolites trigger immunity in Arabidopsis plants. Science 364 (6436), 178–181. 10.1126/science.aau1279 30975887

[B48] Leal A F Suarez D A. Echeverri-Pena O. Y. Albarracin S. L. Almeciga-Diaz C. J. (2022). Olga yaneth echeverri-peña, sonia luz albarracín, carlos javier alméciga-díaz, and ángela johana espejo-MojicaSphingolipids and their role in health and disease in the central nervous system. Adv. Biol. Regul. 85, 100900. 10.1016/j.jbior.2022.100900 35870382

[B49] Lemkul J (2019). From proteins to perturbed Hamiltonians: A suite of tutorials for the GROMACS-2018 molecular simulation package [article v1.0]. Living J. Comput. Mol. Sci. 1 (1). 10.33011/livecoms.1.1.5068

[B50] Liebisch G. Fahy E. Aoki J. Dennis E A. Durand T. Ejsing C S. (2020). Update on LIPID MAPS classification, nomenclature, and shorthand notation for MS-derived lipid structures. J. Lipid Res. 61 (12), 1539–1555. 10.1194/jlr.s120001025 33037133 PMC7707175

[B51] Lindahl A. Spoel V d. (2022). GROMACS 2021.5 manual,” january. 10.5281/zenodo.5849961

[B52] Liu H. Zhao X. Guo M. Liu H. Zheng Z. (2015). Growth and metabolism of beauveria bassiana spores and mycelia. BMC Microbiol. 15 (1), 267–312. 10.1186/s12866-015-0592-4 26581712 PMC4652391

[B53] López C. A. Zofie Sovova F. J. Marrink S. J. de Vries A. H. Marrink S. J. (2013). Martini force field parameters for glycolipids. J. Chem. Theory Comput. 9 (3), 1694–1708. 10.1021/ct3009655 26587629

[B54] Lösel D. M. (1990). Lipids in the structure and function of fungal membranes in biochemistry of cell walls and membranes in fungi, 119–33. Berlin, Heidelberg: Springer.

[B55] Luo F. Wang Q. Yin C. Ge Y. Hu F. Huang B. (2015). Differential metabolic responses of beauveria bassiana cultured in pupae extracts, root exudates and its interactions with insect and plant. J. Invertebr. Pathology 130, 154–164. 10.1016/j.jip.2015.01.003 25584432

[B56] Manocha M. S. (1980). Lipid composition of paracoccidioides brasiliensis: Comparison between the yeast and mycelial forms. Sabouraudia 18 (4), 281–286. 10.1080/00362178085380481 7455860

[B57] Marrink S. J. de Vries A. H. Mark A. E. (2004). Coarse grained model for semiquantitative lipid simulations. J. Phys. Chem. B 108, 750–760. 10.1021/jp036508g

[B58] Marrink S. J. Jelger Risselada H. Yefimov S. Peter Tieleman D. de Vries A. H. (2007). The MARTINI force field: Coarse grained model for biomolecular simulations. J. Phys. Chem. B 111 (27), 7812–7824. 10.1021/jp071097f 17569554

[B59] Mondal D. Malik S. Banerjee P. Kundu N. Debnath A. Sarkar N. (2020). Modulation of membrane fluidity to control interfacial water structure and dynamics in saturated and unsaturated phospholipid vesicles. Langmuir ACS J. Surfaces Colloids 36 (41), 12423–12434. 10.1021/acs.langmuir.0c02736 33035065

[B60] Monje-Galvan V. Klauda J. B. (2015). Modeling yeast organelle membranes and how lipid diversity influences bilayer properties. Biochemistry 54 (45), 6852–6861. 10.1021/acs.biochem.5b00718 26497753

[B61] Monnier N. Cordier M. Dahi A. Santoni V. Guénin S. Clément C. (2020). Semipurified rhamnolipid mixes protect *Brassica napus* against *Leptosphaeria maculans* early infections. Phytopathology 110 (4), 834–842. 10.1094/phyto-07-19-0275-r 31880985

[B62] Monnier N. Furlan A. Botcazon C. Dahi A. Mongelard G. Cordelier S. (2018). Rhamnolipids from *Pseudomonas aeruginosa* are elicitors triggering *Brassica napus* protection against *Botrytis cinerea* without physiological disorders. Front. Plant Sci. 9, 1170. 10.3389/fpls.2018.01170 30135699 PMC6092566

[B63] Monnier N. Furlan A. L. Buchoux S. Deleu M. Dauchez M. Rippa S. (2019). Exploring the dual interaction of natural rhamnolipids with plant and fungal biomimetic plasma membranes through biophysical studies. Int. J. Mol. Sci. 20 (5), 1009. 10.3390/ijms20051009 30813553 PMC6429473

[B64] Moradi S. Amin N. Shahlaei M. (2019). Shedding light on the structural properties of lipid bilayers using molecular dynamics simulation: A review study. RSC Adv. 9 (8), 4644–4658. 10.1039/c8ra08441f 35520151 PMC9060685

[B65] Nasir M. N. Lins L. Crowet J-M. Ongena M. Dorey S. Dhondt-Cordelier S. (2017). Differential interaction of synthetic glycolipids with biomimetic plasma membrane lipids correlates with the plant biological response. Langmuir 33, 9979–9987. 10.1021/acs.langmuir.7b01264 28749675

[B66] Nosé S. (2002). A molecular dynamics method for simulations in the canonical ensemble. Mol. Phys. 100, 191–198. 10.1080/00268970110089108

[B67] Oliva A. Teruel J. A. Aranda F. J. Ortiz. A. (2020). Effect of a dirhamnolipid biosurfactant on the structure and phase behaviour of dimyristoylphosphatidylserine model membranes. Colloids Surfaces. B, Biointerfaces 185, 110576. 10.1016/j.colsurfb.2019.110576 31670001

[B68] Olsen A. S. B. Færgeman N. J. (2017). Sphingolipids: Membrane microdomains in brain development, function and neurological diseases. Open Biol. 7, 170069. 10.1098/rsob.170069 28566300 PMC5451547

[B69] Ortiz A. Teruel J. A. Espuny M. J. Marques A. Manresa A. Aranda F. J. (2006). Effects of dirhamnolipid on the structural properties of phosphatidylcholine membranes. Int. J. Pharm. 325 (1-2), 99–107. 10.1016/j.ijpharm.2006.06.028 16872765

[B70] Páll S. Hess B. (2013). A flexible algorithm for calculating pair interactions on SIMD architectures. Comput. Phys. Commun. 184, 2641–2650. 10.1016/j.cpc.2013.06.003

[B71] Palma-Guerrero J. Lopez-Jimenez J. A. Pérez-Berná A. J. Huang I-C. Jansson H-B. Salinas J. (2010). Membrane fluidity determines sensitivity of filamentous fungi to chitosan. Mol. Microbiol. 75 (4), 1021–1032. 10.1111/j.1365-2958.2009.07039.x 20487294

[B72] Parrinello M. Rahman A. (1981). Polymorphic transitions in single crystals: A new molecular dynamics method. J. Appl. Phys. 52, 7182–7190. 10.1063/1.328693

[B73] Pearlstein R. A. Dickson C. J. Viktor H. (2017). Contributions of the membrane dipole potential to the function of voltage-gated cation channels and modulation by small molecule potentiators. Biochimica Biophysica Acta, Biomembr. 1859 (2), 177–194. 10.1016/j.bbamem.2016.11.005 27836643

[B74] Perczyk P. Wójcik A. Wydro P. Broniatowski M. (2020). The role of phospholipid composition and ergosterol presence in the adaptation of fungal membranes to harsh environmental conditions-membrane modeling study. Biochimica Biophysica Acta, Biomembr. 1862 (2), 183136. 10.1016/j.bbamem.2019.183136 31751523

[B75] Phoenix D. A. Harris F. Mura M. Dennison S. R. (2015). The increasing role of phosphatidylethanolamine as a lipid receptor in the action of host defence peptides. Prog. Lipid Res. 59, 26–37. 10.1016/j.plipres.2015.02.003 25936689

[B76] Piironen V. Lindsay D. G. Miettinen T. A. Toivo J. Lampi A-M. (2000). Plant sterols: Biosynthesis, biological function and their importance to human nutrition. J. Sci. Food Agric. 80. 10.1002/(sici)1097-0010(20000515)80:7<939:aid-jsfa644>3.0.co;2-c

[B77] Pitt J. I. Hocking A. D. (2009). Fresh and perishable foods. Fungi and food spoilage, 383–400. Boston, MA: Springer.

[B78] Project I. (2020). Inkscape. Massachusetts, USA: Free Software Foundation Boston.

[B79] Ramos-Martín F. Herrera-León C. Antonietti V. Pascal S. Sarazin C. D’Amelio N. (2022). The potential of antifungal peptide sesquin as natural food preservative. Biochimie 51, 60. 10.1016/j.biochi.2022.03.015 35395327

[B80] Ramos-Martín F. D’Amelio A. (2022). Biomembrane lipids: When physics and Chemistry join to shape biological activity. Biochimie 203, 118–138. 10.1016/j.biochi.2022.07.011 35926681

[B81] Randhawa S. Kamaljeet K. PattanathuRahman. K. S. M. (2014). Rhamnolipid biosurfactants-past, present, and future scenario of global market. Front. Microbiol. 5, 454. 10.3389/fmicb.2014.00454 25228898 PMC4151382

[B82] Ranf S. (2017). Sensing of molecular patterns through cell surface immune receptors. Curr. Opin. Plant Biol. 38, 68–77. 10.1016/j.pbi.2017.04.011 28501024

[B83] Robineau M. Le Guenic S. Sanchez L. Chaveriat L. Lequart V. Joly N. (2020). Synthetic mono-rhamnolipids display direct antifungal effects and trigger an innate immune response in tomato against Botrytis cinerea. Molecules 25, 3108. 10.3390/molecules25143108 32650401 PMC7397090

[B84] Sanchez L. Courteaux B. Hubert J. Kauffmann S. Jean-Hugues R. Clément C. Baillieul F. (2012). Rhamnolipids elicit defense responses and induce disease resistance against biotrophic, hemibiotrophic, and necrotrophic pathogens that require different signaling pathways in Arabidopsis and highlight a central role for salicylic acid. Plant Physiol. 160 (3), 1630–1641. 10.1104/pp.112.201913 22968829 PMC3490604

[B85] Sánchez M. Aranda F. J. Teruel J. A. Espuny M. J. Marques A. Manresa A. (2010). Ana marqués, angeles manresa, and antonio OrtizPermeabilization of biological and artificial membranes by a bacterial dirhamnolipid produced by Pseudomonas aeruginosa. J. Colloid Interface Sci. 341 (2), 240–247. 10.1016/j.jcis.2009.09.042 19837413

[B86] Sánchez M. Aranda F. J. Teruel J. A. Ortiz. A. (2009). Interaction of a bacterial dirhamnolipid with phosphatidylcholine membranes: A biophysical study. Chem. Phys. Lipids 161, 51–55. 10.1016/j.chemphyslip.2009.06.145 19580793

[B87] Sánchez M. Teruel J. A. Espuny M. J. Marques A. Aranda F. J. Manresa A. (2006). Ana marqués, francisco J. Aranda, angeles manresa, and antonio OrtizModulation of the physical properties of dielaidoylphosphatidylethanolamine membranes by a dirhamnolipid biosurfactant produced by Pseudomonas aeruginosa. Chem. Phys. Lipids 142 (1-2), 118–127. 10.1016/j.chemphyslip.2006.04.001 16678142

[B88] Santos F. C. Marquês J. T. Bento-Oliveira A. Rodrigode Almeida F. M. (2020). Sphingolipid-enriched domains in fungi. FEBS Lett. 594 (22), 3698–3718. 10.1002/1873-3468.13986 33141925

[B89] SantosDanyelle Khadydja F. Rufino R. D. Luna J. M. Santos V. A. Sarubbo L. A. (2016). Biosurfactants: Multifunctional biomolecules of the 21st century. Int. J. Mol. Sci. 17 (3), 401. 10.3390/ijms17030401 26999123 PMC4813256

[B90] Schellenberger R. Crouzet J. Nickzad A. Shu L.-J. Alexander K. (2021). Bacterial rhamnolipids and their 3-hydroxyalkanoate precursors activate *Arabidopsis* innate immunity through two independent mechanisms. Proc. Natl. Acad. Sci. U. S. A. 118 (39), e2101366118. 10.1073/pnas.2101366118 34561304 PMC8488661

[B91] Sezgin E. Levental I. Mayor S. Eggeling C. (2017). The mystery of membrane organization: Composition, regulation and roles of lipid rafts. Nat. Rev. Mol. Cell Biol. 18 (6), 361–374. 10.1038/nrm.2017.16 28356571 PMC5500228

[B92] Sha R. Qin M. (2016). Antifungal activity of rhamnolipids against dimorphic fungi. J. General Appl. Microbiol. 62 (5), 233–239. 10.2323/jgam.2016.04.004 27666589

[B93] Smith P. Lorenz C. D. (2021). LiPyphilic: A Python toolkit for the analysis of lipid membrane simulations. J. Chem. Theory Comput. 17 (9), 5907–5919. 10.1021/acs.jctc.1c00447 34450002

[B94] Snowdon A. L. (1990). Post-harvest diseases and disorders of fruits and vegetables: Volume 1: General introduction and fruits. Taylor and Francis.

[B95] Stanghellini M. E. Miller. R. M. (1997). Biosurfactants: Their identity and potential efficacy in the biological control of zoosporic plant pathogens. Plant Dis. 81 (1), 4–12. 10.1094/pdis.1997.81.1.4 30870944

[B96] Sud M. Fahy E. Cotter D. Brown A. Dennis E. A. Glass C. K. (2006). Lmsd: LIPID MAPS structure Database. Nucleic Acids Res. 35 (1), D527–D532. 10.1093/nar/gkl838 17098933 PMC1669719

[B97] Thevissen K. Ferket K. K. A. François I. E. J. A. Cammue B. P. A. (2003). Interactions of antifungal plant defensins with fungal membrane components. Peptides 24 (11), 1705–1712. 10.1016/j.peptides.2003.09.014 15019201

[B98] Valitova J. N. Sulkarnayeva A. G. Minibayeva F. V. (2016). Plant sterols: Diversity, biosynthesis, and physiological functions. Biokhimiia 81 (8), 819–834. 10.1134/s0006297916080046 27677551

[B99] Vance D. (2000). Cholesterol in the year 2000. Biochimica Biophysica Acta, Mol. Cell Biol. Lipids 1529 (1-3), 1–8. 10.1016/s1388-1981(00)00133-5 11111073

[B100] Varnier A.-L. Sanchez L. Vatsa P. Boudesocque L. Garcia-Brugger A. Rabenoelina F. (2009). Bacterial rhamnolipids are novel MAMPs conferring resistance to Botrytis cinerea in grapevine. Plant, Cell and Environ. 32 (2), 178–193. 10.1111/j.1365-3040.2008.01911.x 19021887

[B101] Vecer J. Vesela P. Jan M. Herman P. (2014). Sphingolipid levels crucially modulate lateral microdomain organization of plasma membrane in living yeast. FEBS Lett. 588 (3), 443–449. 10.1016/j.febslet.2013.11.038 24333335

[B102] Volkman J. K. (2003). Sterols in microorganisms. Appl. Microbiol. Biotechnol. 60 (5), 495–506. 10.1007/s00253-002-1172-8 12536248

[B21] van Eerden F. J. V. Djurrede Jong H. de Vries A. H. Wassenaar T. A. Marrink S. J. (2015). Characterization of thylakoid lipid membranes from cyanobacteria and higher plants by molecular dynamics simulations. Biochimica Biophysica Acta 1848 (6), 1319–1330. 10.1016/j.bbamem.2015.02.025 25749153

[B103] Wang L. (2012). Measurements and implications of the membrane dipole potential. Annu. Rev. Biochem. 81, 615–635. 10.1146/annurev-biochem-070110-123033 22443933

[B104] Wassenaar T. A. Ingólfsson H. I. Böckmann R. A. Tieleman D. P. Marrink S. J. (2015). Computational lipidomics with insane: A versatile tool for generating custom membranes for molecular simulations. J. Chem. Theory Comput. 11 (5), 2144–2155. 10.1021/acs.jctc.5b00209 26574417

[B105] Weete J. D. (1980). Glycerophospholipids. In lipid biochemistry of fungi and other organisms, 157–79. Boston, MA: Springer.

[B106] Yan F. Hu H. Lu L. Zheng X. (2016). Rhamnolipids induce oxidative stress responses in cherry tomato fruit to alternaria alternata. Pest Manag. Sci. 72 (8), 1500–1507. 10.1002/ps.4177 26506951

[B107] Yu M. Cui Y. Zhang X. Li R. Lin J. (2020a). Organization and dynamics of functional plant membrane microdomains. Cell. Mol. Life Sci. CMLS 77 (2), 275–287. 10.1007/s00018-019-03270-7 31422442 PMC11104912

[B108] Zakharova A. A. Efimova S. S. Malev V. V. Ostroumova O. S. (2019). Fengycin induces ion channels in lipid bilayers mimicking target fungal cell membranes. Sci. Rep. 9 (1), 16. 10.1038/s41598-019-52551-5 31690786 PMC6831686

[B109] Zhao C. Tombola F. (2021). Voltage-gated proton channels from fungi highlight role of peripheral regions in channel activation. Commun. Biol. 4 (1), 261. 10.1038/s42003-021-01792-0 33637875 PMC7910559

