# Supplementary material for: The effect of rhamnolipids on fungal membrane models as described by their interactions with phospholipids and sterols: An in silico study
Source: Front Chem. 2023 Feb 21;11:1124129. doi: 10.3389/fchem.2023.1124129 (PMC9989204; doi:10.3389/fchem.2023.1124129)
Supplement: Supplementary file 1 [file DataSheet1.PDF]

frontiers

Supplementary Material

The effect of rhamnolipids on fungal membrane models as described by their interactions with phospholipids and sterols: an *in-silico* study

Nely Rodríguez-Moraga[1], Francisco Ramos-Martín[1*], Sébastien Buchoux[1], Sonia Rippa[2], Nicola D'Amelio[1] and Catherine Sarazin[1*]

[1] Unité de Génie Enzymatique et Cellulaire UMR 7025 CNRS, Université de Picardie Jules Verne, Amiens, 80039, France

[2] Unité de Génie Enzymatique et Cellulaire, CNRS UMR 7025, Sorbonne Universités, Université de Technologie de Compiègne, Compiègne, France

* To whom correspondence should be addressed. Tel: +33 3 22 82 7473; Fax: +33 3 22 82 75 95; Email: catherine.sarazin@u-picardie.fr , francisco.ramos@u-picardie.fr .

Mono-RL CG Topology

**Bonds**	**r_0 (nm)**	**k_r (kJ mol^{-1} nm^{-2})**	**Angles**	**Θ_0 (deg)**	**k_Θ (kJ mol^{-1})**	**Exclusions**
**(1)** 1-2	0.279	38000	**(1)** 1-2-3	64.0	50.0	3-5
**(2)** 1-3	0.287	20000	**(2)** 1-3-2	61.0	50.0	1-4
**(3)** 2-3	0.264	44000	**(3)** 1-3-4	131.0	25.0	2-4
**(4)** 3-4	0.380	3000	**(4)** 2-1-3	55.0	30.0	
**(5)** 4-5	0.420	3000	**(5)** 2-3-4	160.0	50.0	
**(6)** 4-6	0.400	1250	**(6)** 3-4-5	160.0	15.0	
**(7)** 5-8	0.400	1250	**(7)** 4-6-7	150.0	15.0	
**(8)** 6-7	0.400	1250	**(8)** 5-8-9	150.0	15.0	
**(9)** 8-9	0.400	1250				

Table S1: Mono-RL chosen CG topology.

Di-RL CG Topology

Bonds	r_0 (nm)	k_r (kJ mol^{-1} nm^{-2})	Angles	Θ_0 (deg)	k_Θ (kJ mol^{-1})	Exclusions
**(1)** 1-2	0.30	25000	**(1)** 1-2-3	48.0	50.0	6-8
**(2)** 1-3	0.26	25000	**(2)** 1-3-4	122.0	50.0	
**(3)** 2-3	0.30	15000	**(3)** 2-3-4	165.0	50.0	
**(4)** 3-4	0.29	15000	**(4)** 3-4-5	113.0	50.0	
**(5)** 4-5	0.29	15000	**(5)** 3-4-6	50.0	50.0	
**(6)** 4-6	0.29	15000	**(6)** 4-5-6	58.0	50.0	
**(7)** 5-6	0.29	15000	**(7)** 4-6-5	62.0	50.0	
**(8)** 6-7	0.36	3500	**(8)** 4-6-7	132.0	50.0	
**(9)** 7-8	0.42	3500	**(9)** 5-6-7	101.0	50.0	
**(10)** 7-9	0.42	1500	**(10)** 6-7-8	130.0	10.0	
**(11)** 9-10	0.42	1500	**(11)** 6-7-9	50.0	50.0	
**(12)** 8-11	0.42	1500	**(12)** 7-9-10	137.0	10.0	
**(13)** 11-12	0.42	1500	**(13)**8-11-12	150.0	50.0	

Table S2: Di-RL chosen CG topology.

	Model 1		Model 2	
	Control	With RLs	Control	With RLs
**Apl (nm^2)**	0.455 ± 0.001	0,454 ± 0,001	0.466 ± 0.002	0.463 ±0.001
**Thickness (nm)**	3.879 ± 0.008	3,848 ± 0,008	3.812 ± 0.007	3.801 ± 0.007
**Membrane area (nm^2)**	1229 ± 3	1226 ± 3	1258 ± 4	1298 ± 3
**Lipid diffusion (10^{-7} cm^2 /s)**				
POPC<br>DOPC	1.07 ± 0,07<br>--	1.10 ± 0.02<br>--	--<br>1.15 ± 0.03	--<br>1.07 ± 0.06
POPE<br>DOPE	1.05 ± 0,04<br>--	1.06 ± 0.01<br>--	1.06 ± 0.06<br>1.04 ± 0.01	1.03 ± 0.00<br>1.05 ± 0.05
PIP_1<br>POPI	0.97 ± 0,06<br>--	1.06 ± 0.12<br>--	--<br>1.03 ± 0.01	--<br>1.04 ± 0.10
POPS<br>DOPS	1.04 ± 0,01<br>--	0.93 ± 0.09<br>--	1.03 ± 0.02<br>1.01 ± 0.07	1.04 ± 0.04<br>1.17 ± 0.12
DOPA	--	--	1.11 ± 0.00	1.08 ± 0.07
ERG	1.19 ± 0,02	1.15 ± 0.06	1.23 ± 0.04	1.24 ± 0,00
Mono-RL		1.00 ± 0.20		1.37 ± 0.02
Di-RL		1.08 ± 0.13		1.14 ± 0.10
**Flip-flops (s^{-1})**				
Ergosterol	4.45×10^5	5.57×10^5	6.26×10^5	6.83×10^5

Table S3. Structural and dynamical parameters of CG model membranes in the absence and in the presence of RLs.

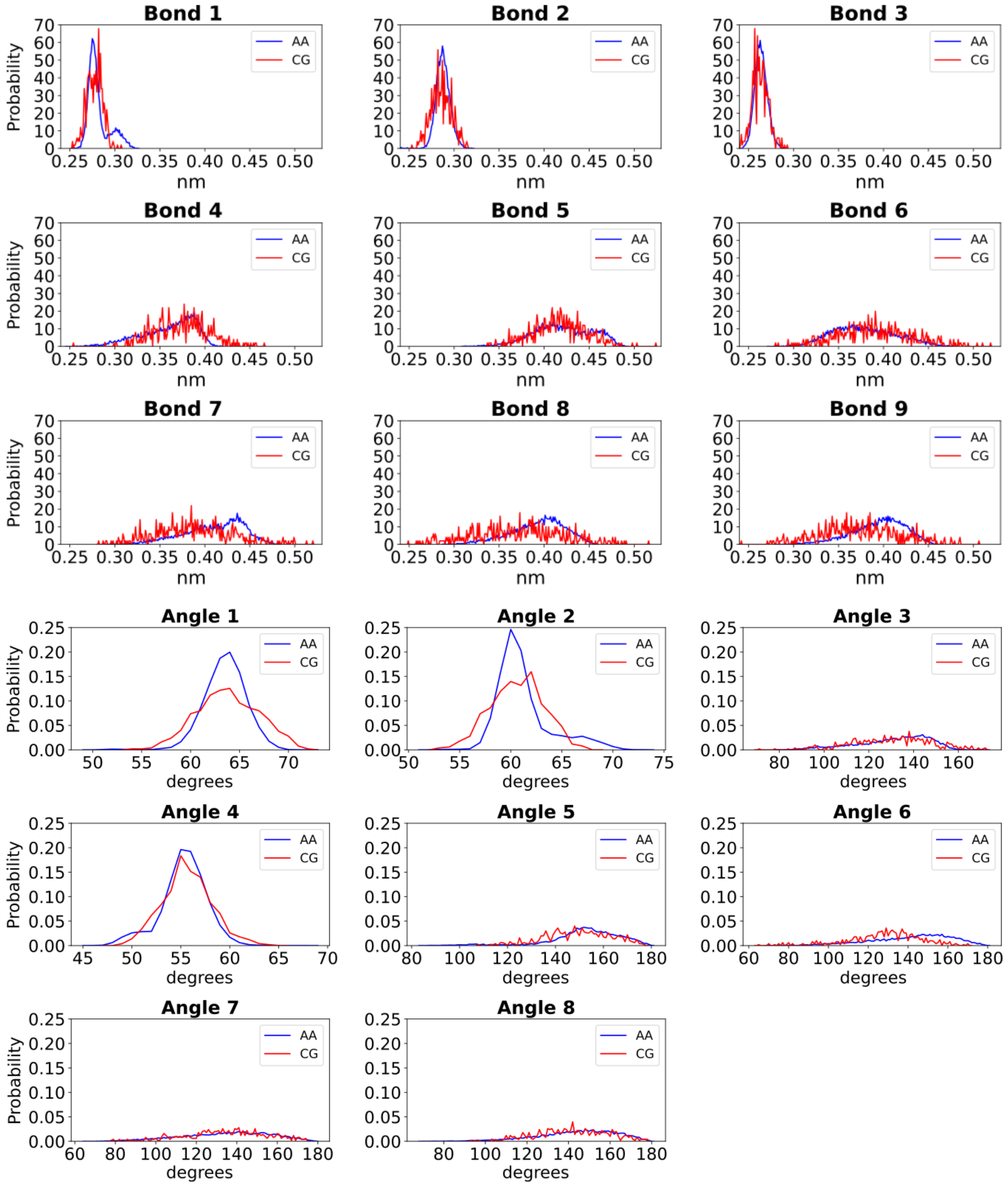

Figure S1. AA vs CG bond, angle and dihedral angle distributions using the chosen mono-RL CG topology (simulated in a POPC membrane).

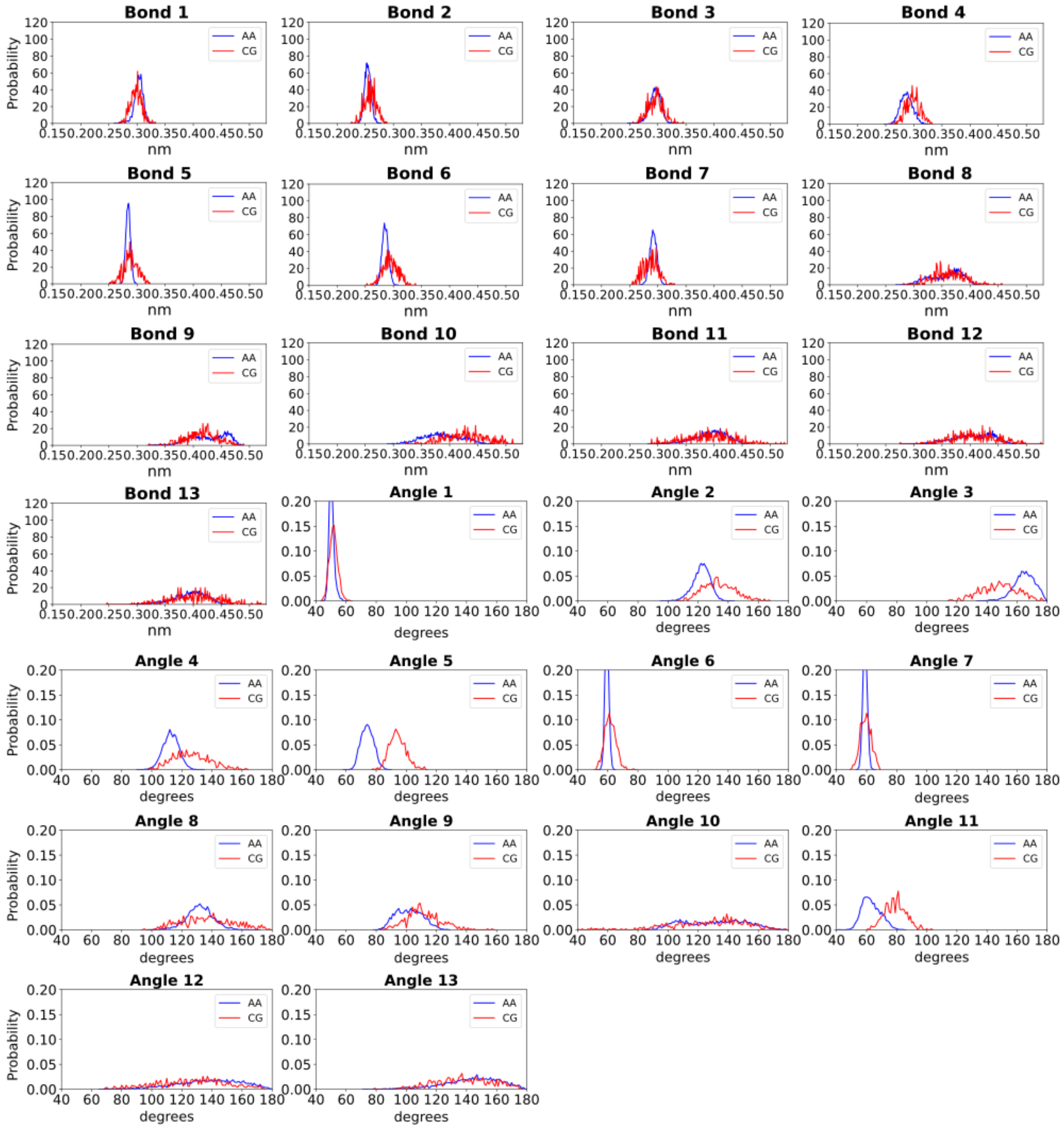

Figure S2. AA vs CG bond, angle and dihedral angle distributions using the chosen di-RL CG topology (simulated in a POPC membrane).

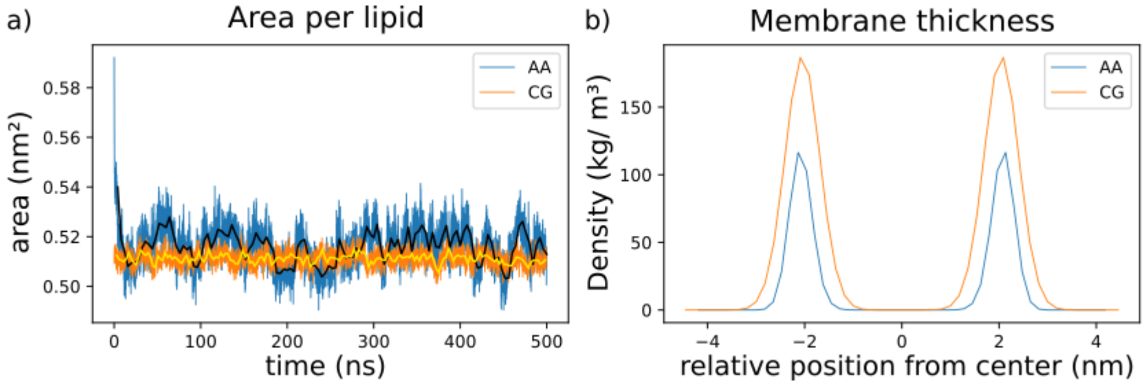

Figure S3. a) Area per lipid and b) membrane thickness for a POPC/POPG/ergosterol (52/23/25) model membrane, described with the chosen topology. Atomic and coarse grained data are shown in blue and orange respectively. For ease of visualisation, the 4 ns rolling average of the area per lipid is shown in black and yellow for AA and CG respectively.

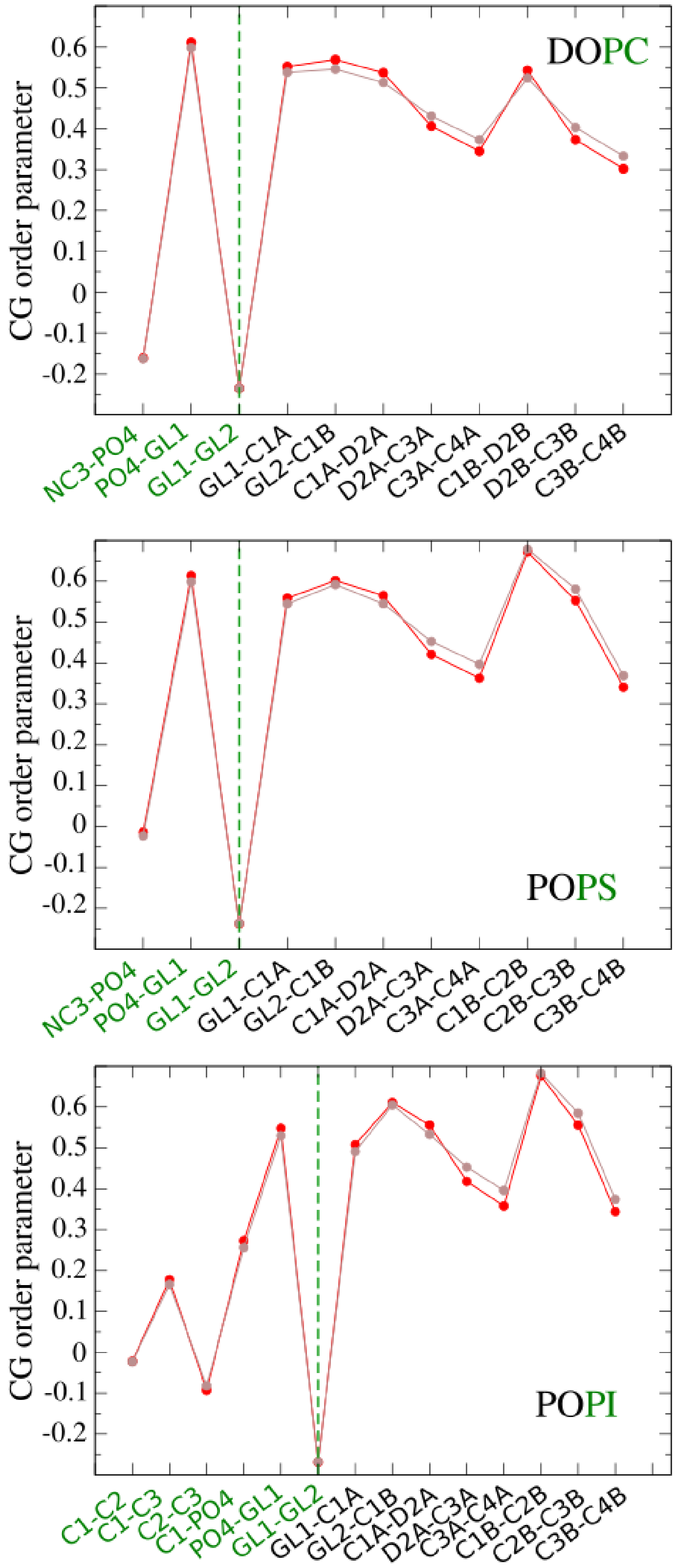

Figure S4. CG order parameters of some phospholipids from model 2 (DOPA/DOPC/DOPE/POPI/POPS/DOPS/Ergosterol 5/12/9/4/12/14/4/40) with and without RLs (shown in red and brown, respectively). Bonds implied in modelled polar heads are shown in green (see Figure 2D for CG bead names).

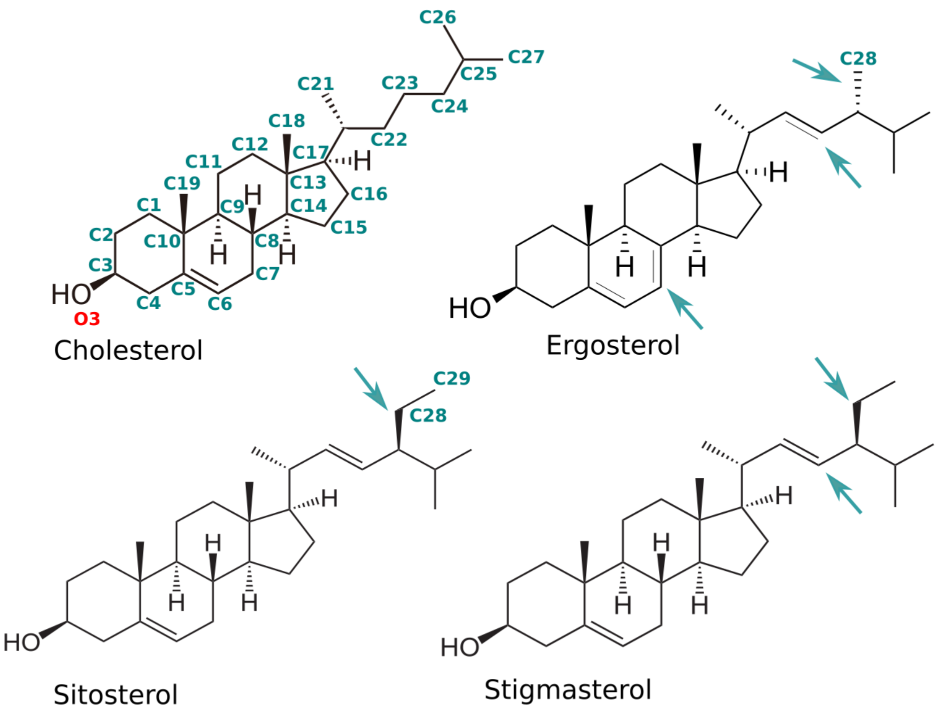

Figure S5. Schemas of cholesterol, ergosterol, sitosterol and stigmasterol molecules highlighting their differences.

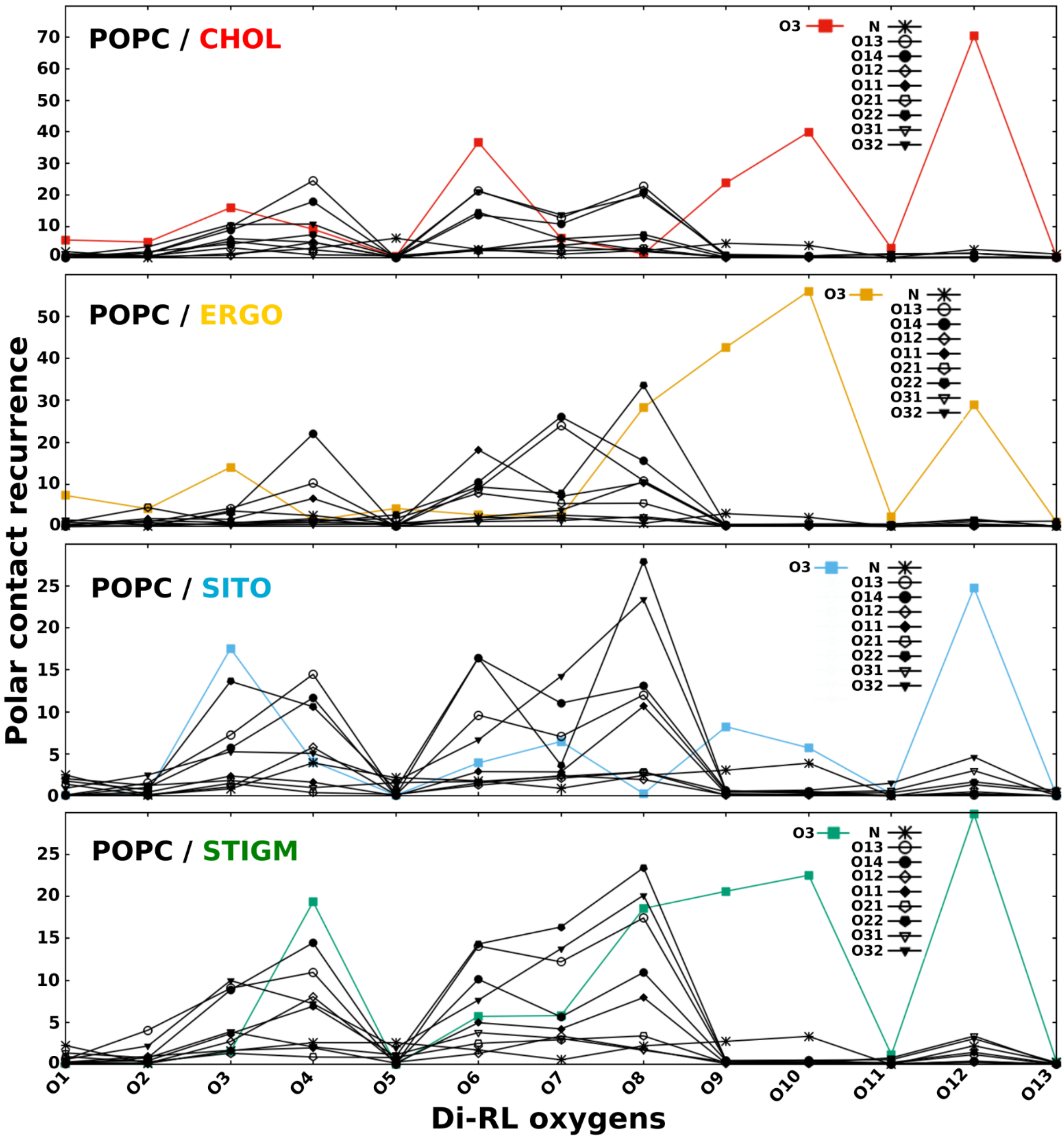

Figure S6. Recurrence of close contacts between polar atoms of di-RLs and those of phospholipids, indicating the formation of H-bonds or salt bridges. Membrane models: single lipid POPC and mixed models of POPC:sterol (70:30)

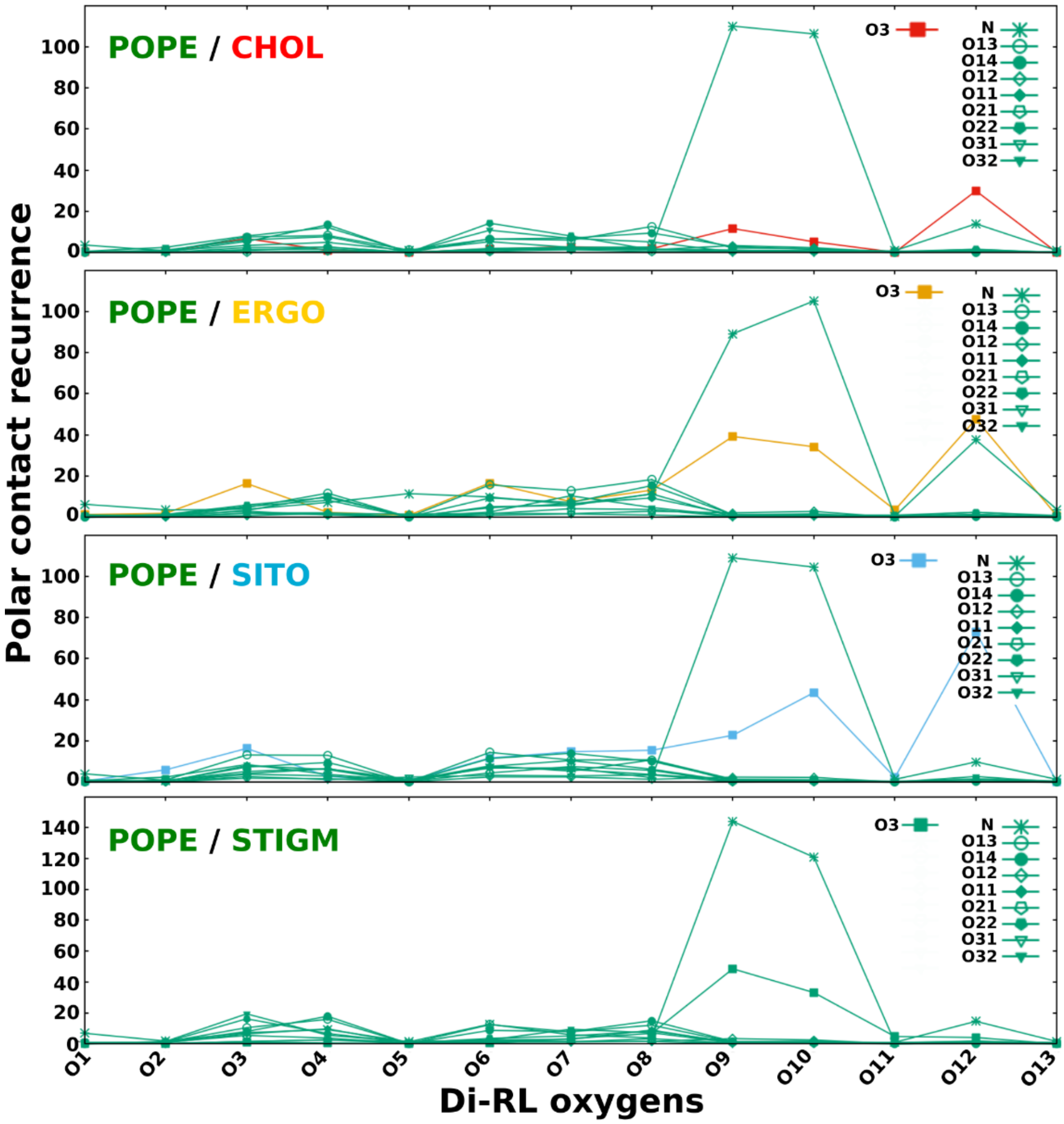

Figure S7. Recurrence of close contacts between polar atoms of di-RLs and those of phospholipids, indicating the formation of H-bonds or salt bridges. Membrane models: single lipid POPE and mixed models of POPE:sterol (70:30)

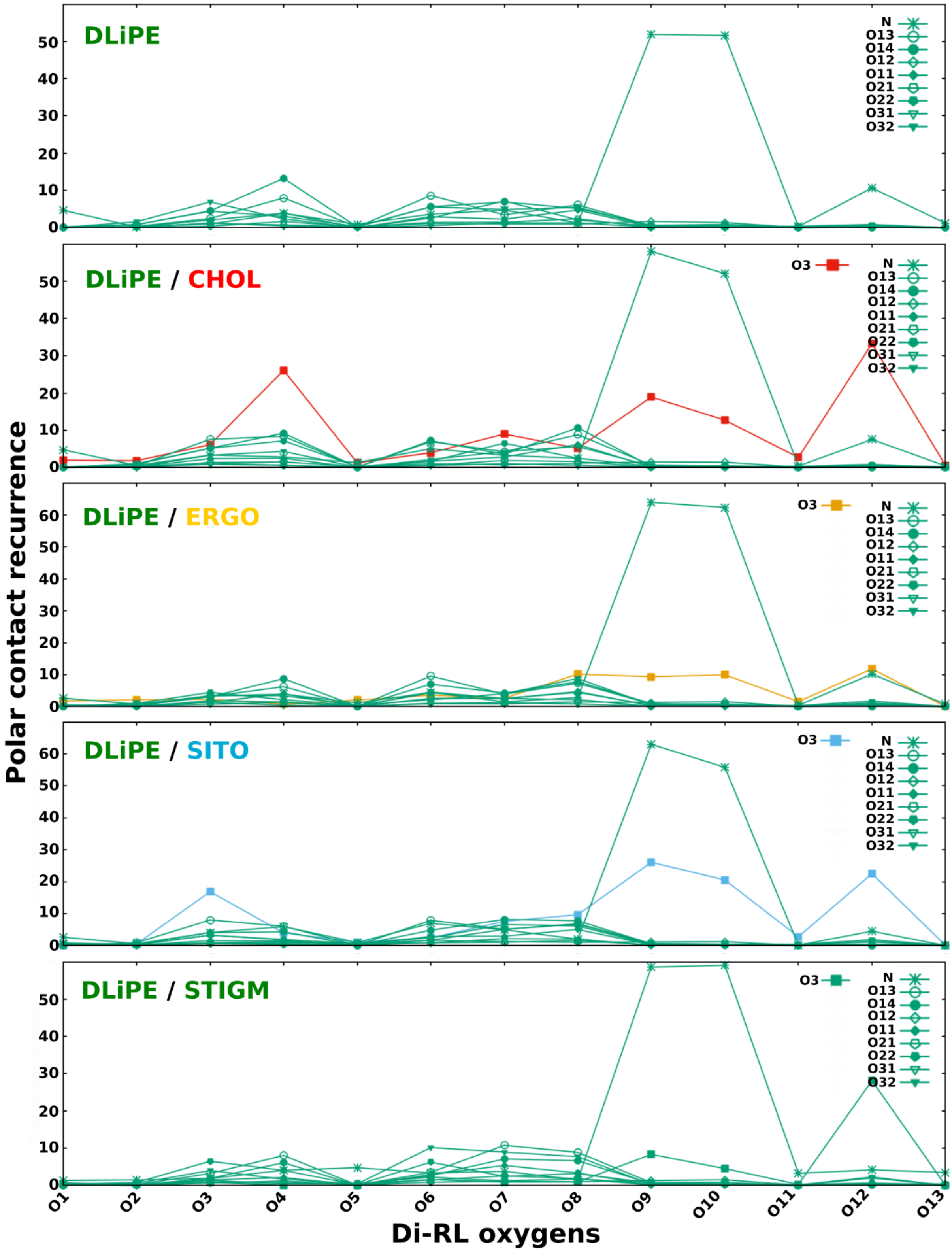

Figure S8. Recurrence of close contacts between polar atoms of di-RLs and those of phospholipids, indicating the formation of H-bonds or salt bridges. Membrane models: single lipid DLiPE and mixed models of DLiPE:sterol (70:30)

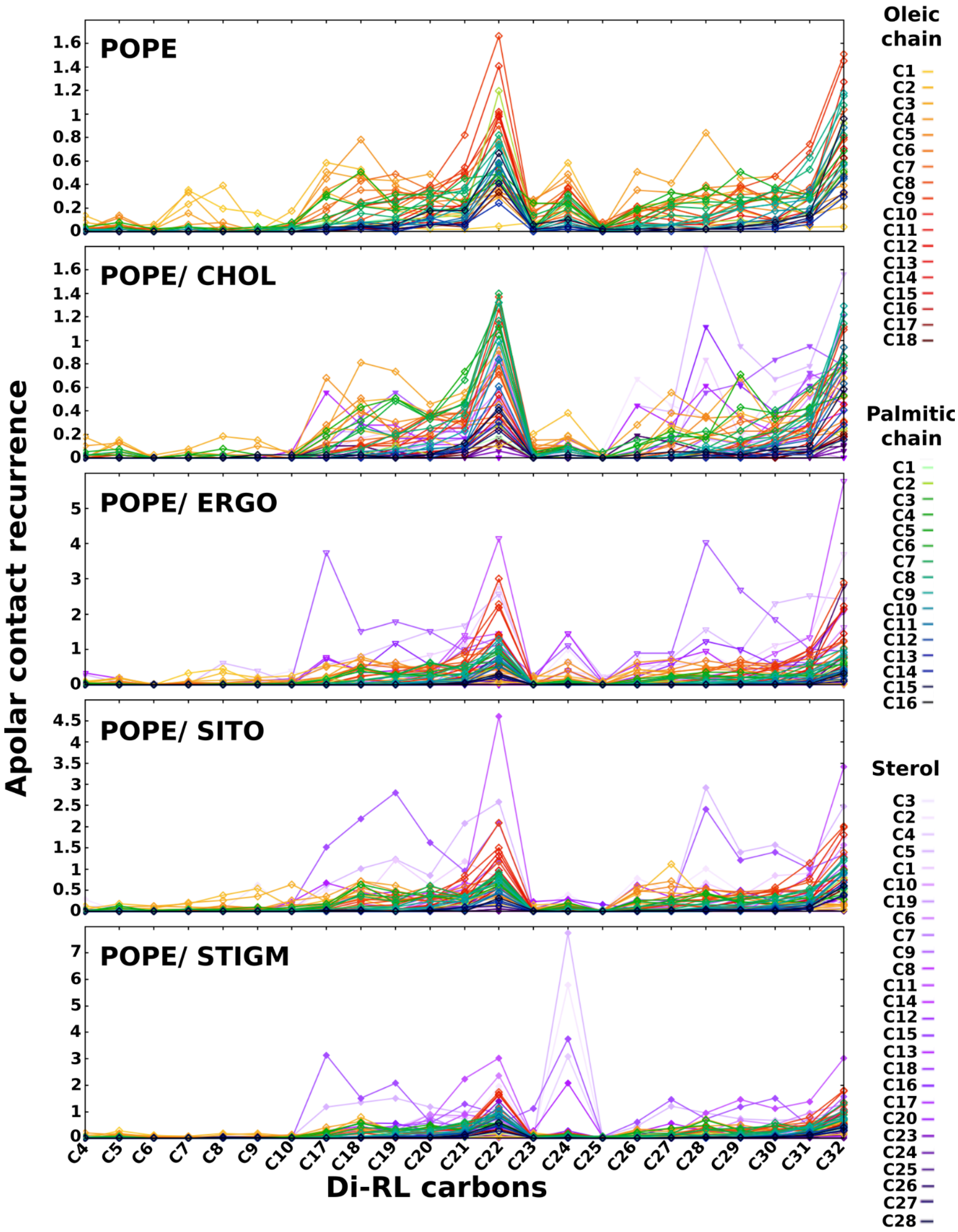

Figure S9. Recurrence of close contacts between apolar atoms of di-RLs and those of phospholipids, indicating the formation of van der Waals contacts. POPE; POPE:sterol (70:30 molar ratios). Colour tonality reflects the distance from the headgroup. Carbons are numbered in order from the carbonyl group for palmitic and oleic chains. Sterols’ carbon numbering is depicted in Figure S5.

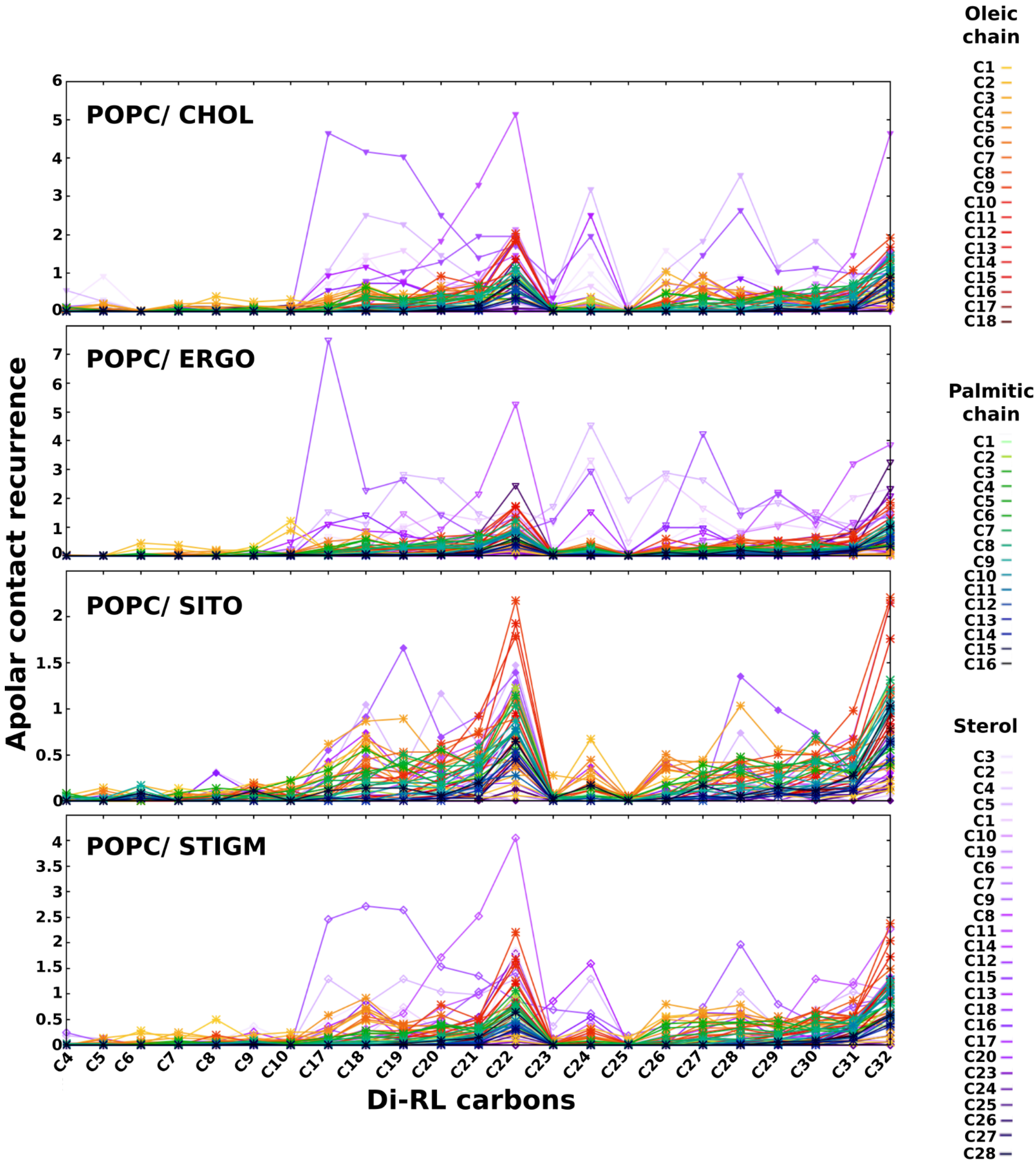

Figure S10. Recurrence of close contacts between apolar atoms of di-RLs and those of phospholipids, indicating the formation of van der Waals contacts. POPC; POPC:sterol (70:30 molar ratios). Colour tonality reflects the distance from the headgroup. Carbons are numbered in order from the carbonyl group for palmitic and oleic chains. Sterols' carbon numbering is depicted in Figure S5.

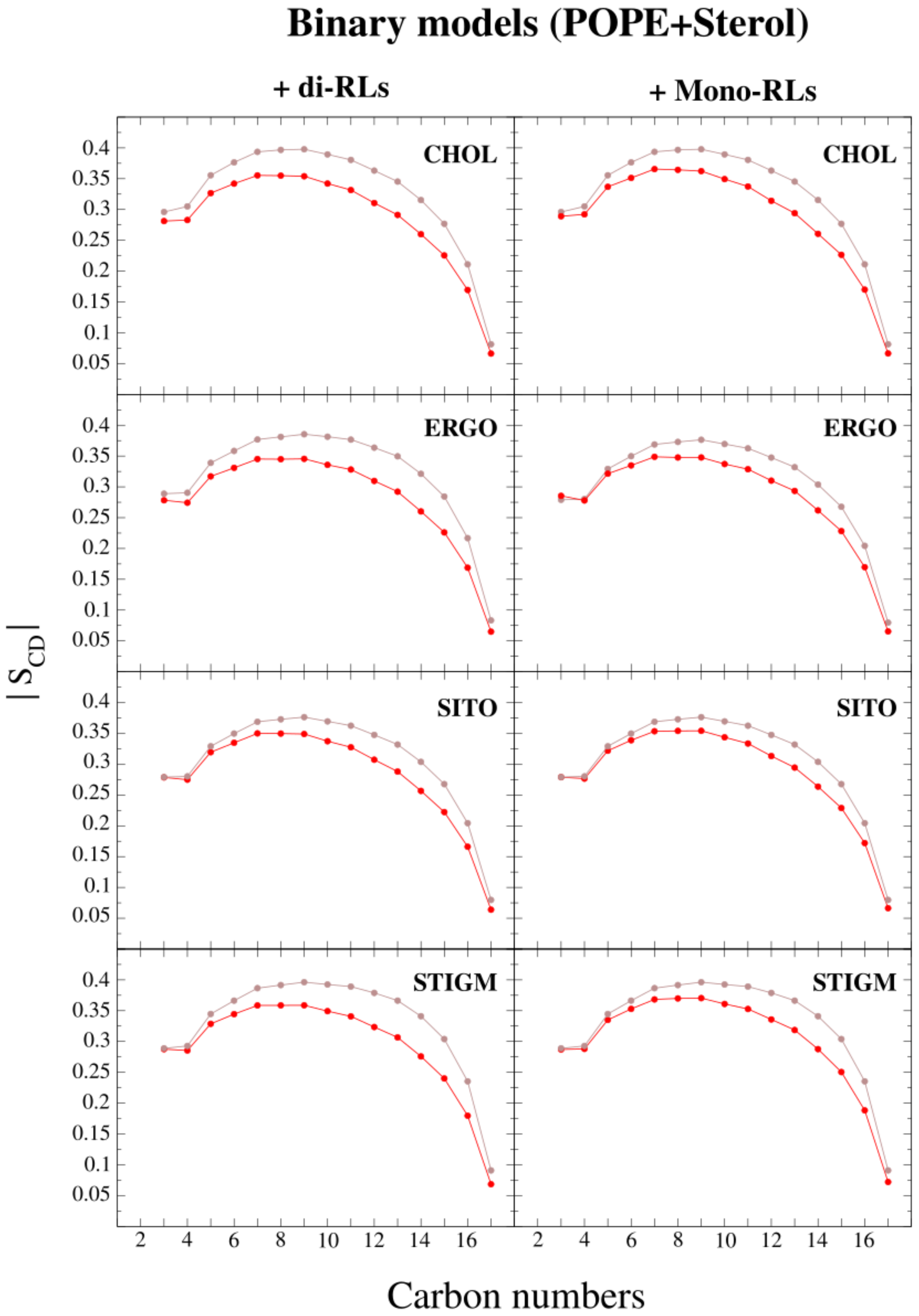

Figure S11. The deuterium order parameters versus the carbon position of the sn-1 acyl chain of binary model membranes POPE/CHOL, POPE/ERGO, POPE/SITO and POPE/STIGM (molar ratios 70:30) either with 5 di-RLs or with 5 mono-RL. Control membranes (without RLs) are shown brown while RLs containing membranes are shown in red. Error bars represent the standard deviation of the last averaged 250ns (smaller than the symbol size in all cases).

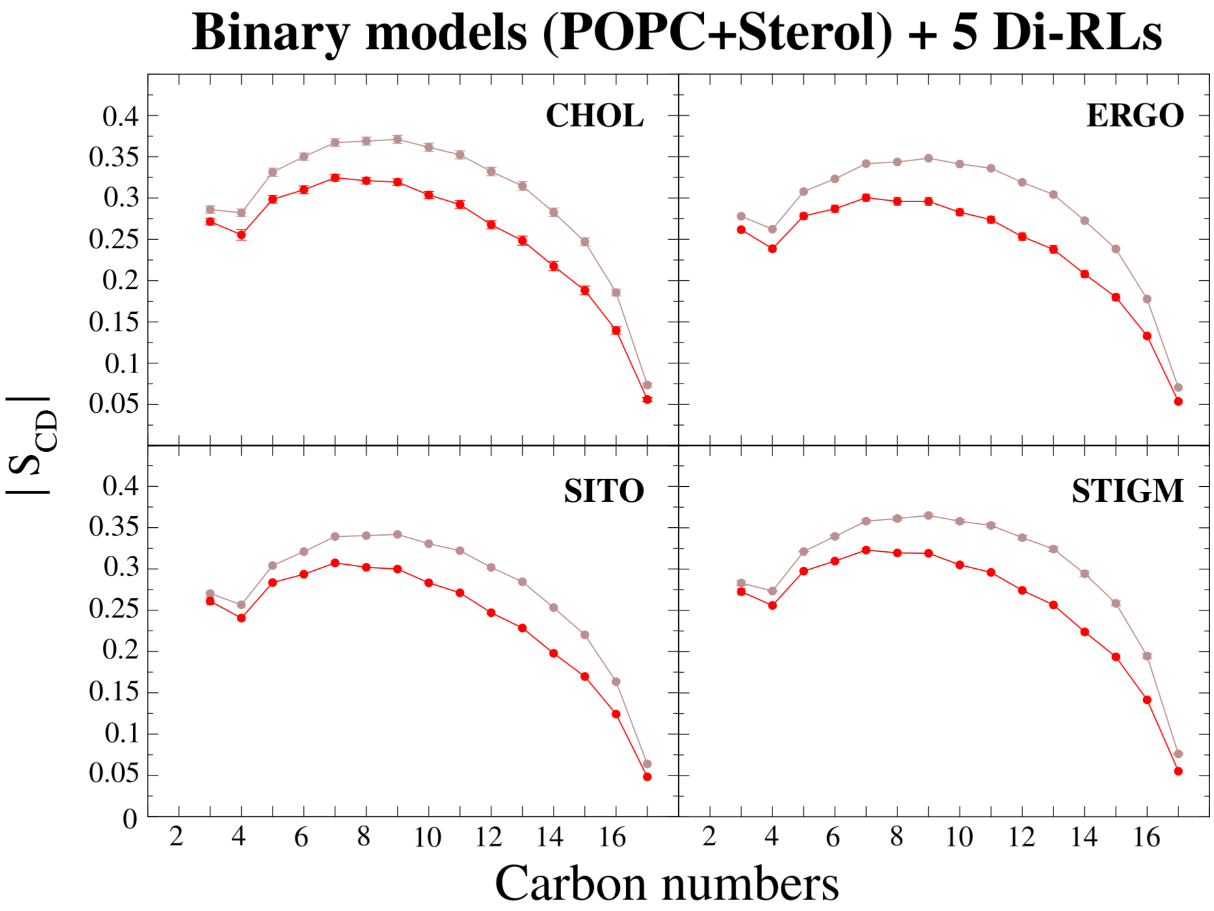

Figure S12. The deuterium order parameters versus the carbon position of the sn-1 acyl chain of binary model membranes POPC/CHOL, POPC/ERGO, POPC/SITO and POPC/STIGM (molar ratios 70:30) with 5 di-RLs. Control membranes (without RLs) are shown brown while RLs containing membranes are shown in red. Error bars represent the standard deviation of the last averaged 250ns (smaller than the symbol size in all cases).

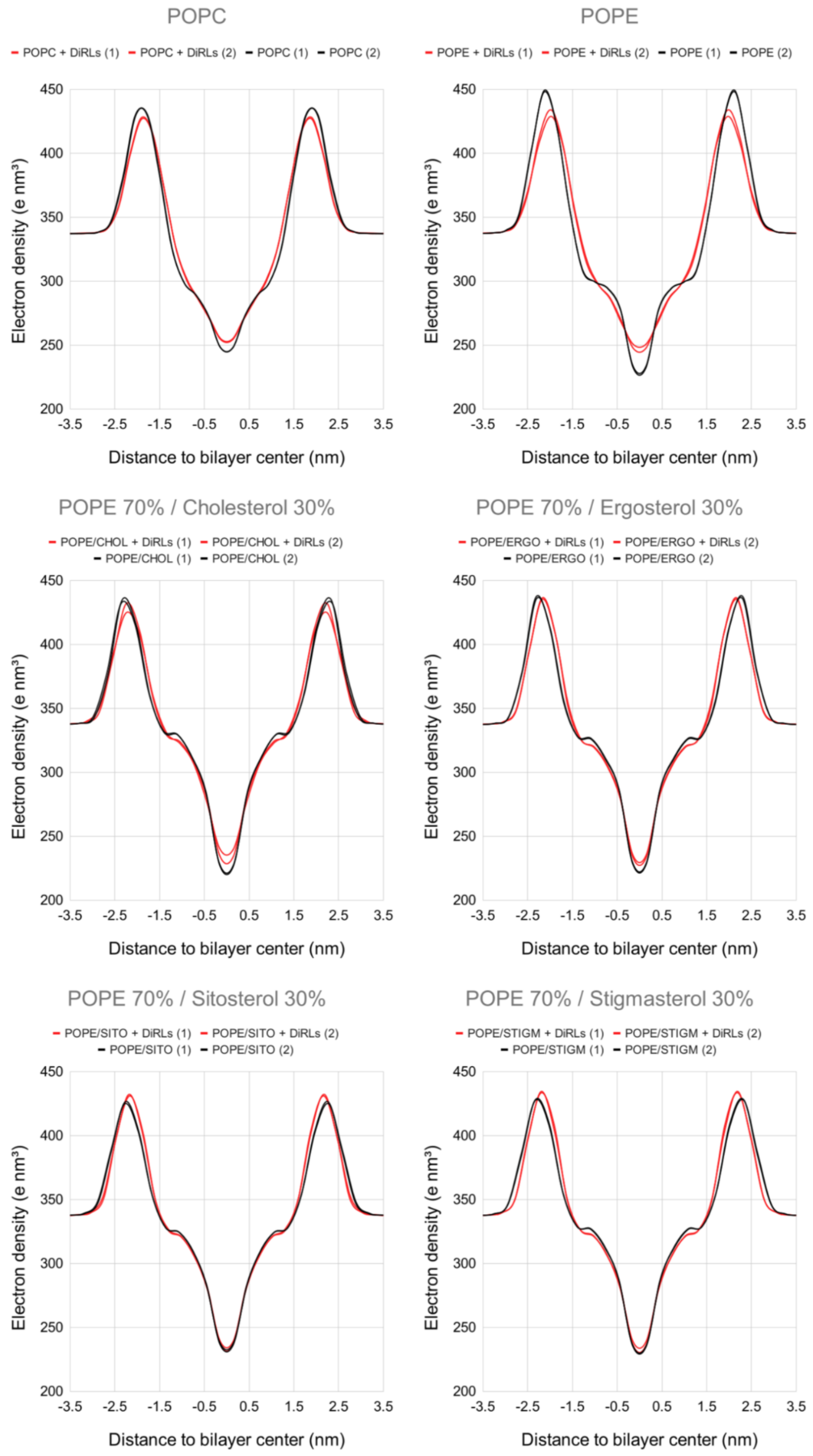

Figure S13. Electron density profiles for bilayers of various lipid compositions as calculated from MD simulations with and without di-RLs.

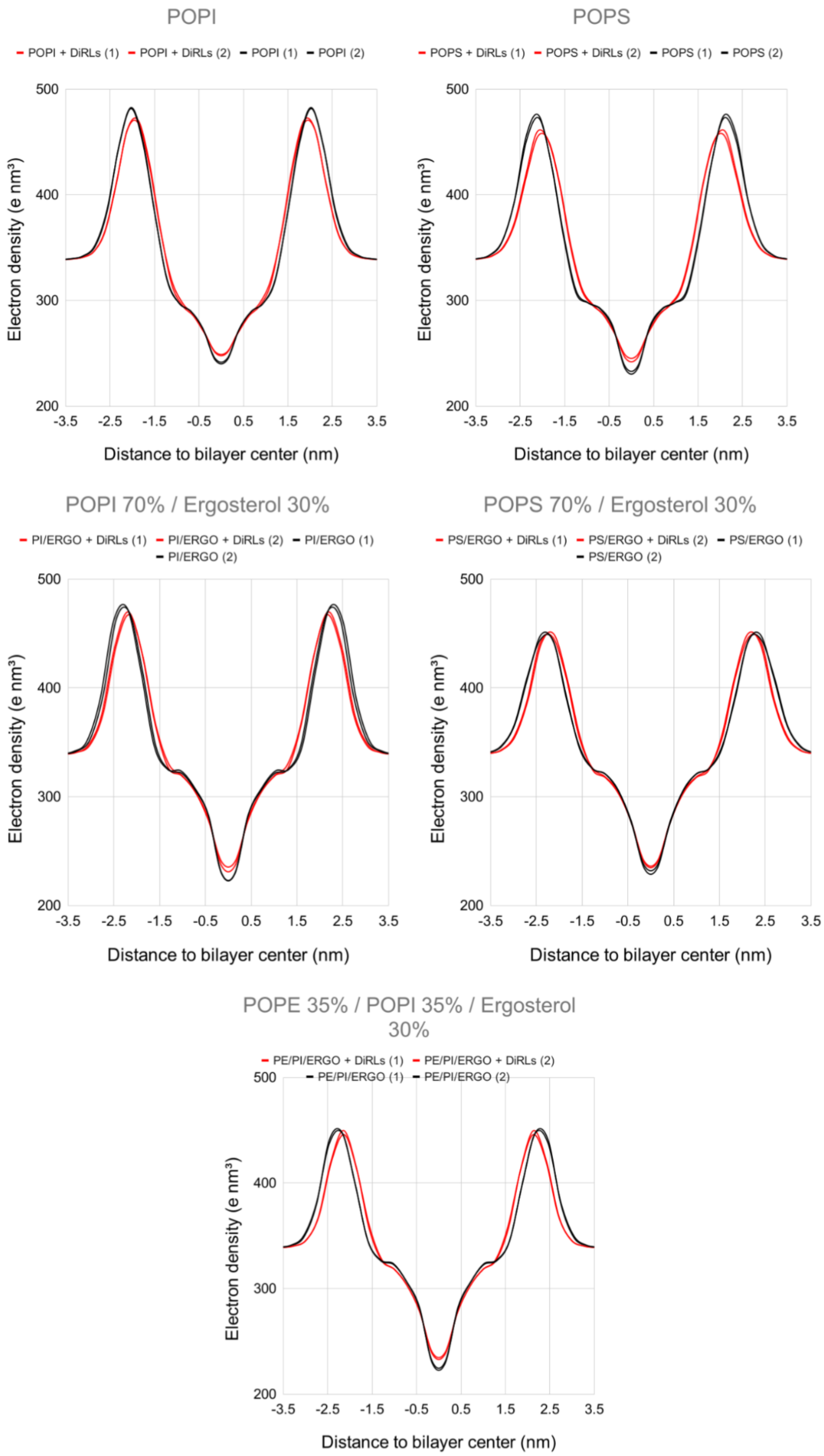

Figure S14. Electron density profiles for bilayers of various lipid compositions as calculated from MD simulations with and without RLs.

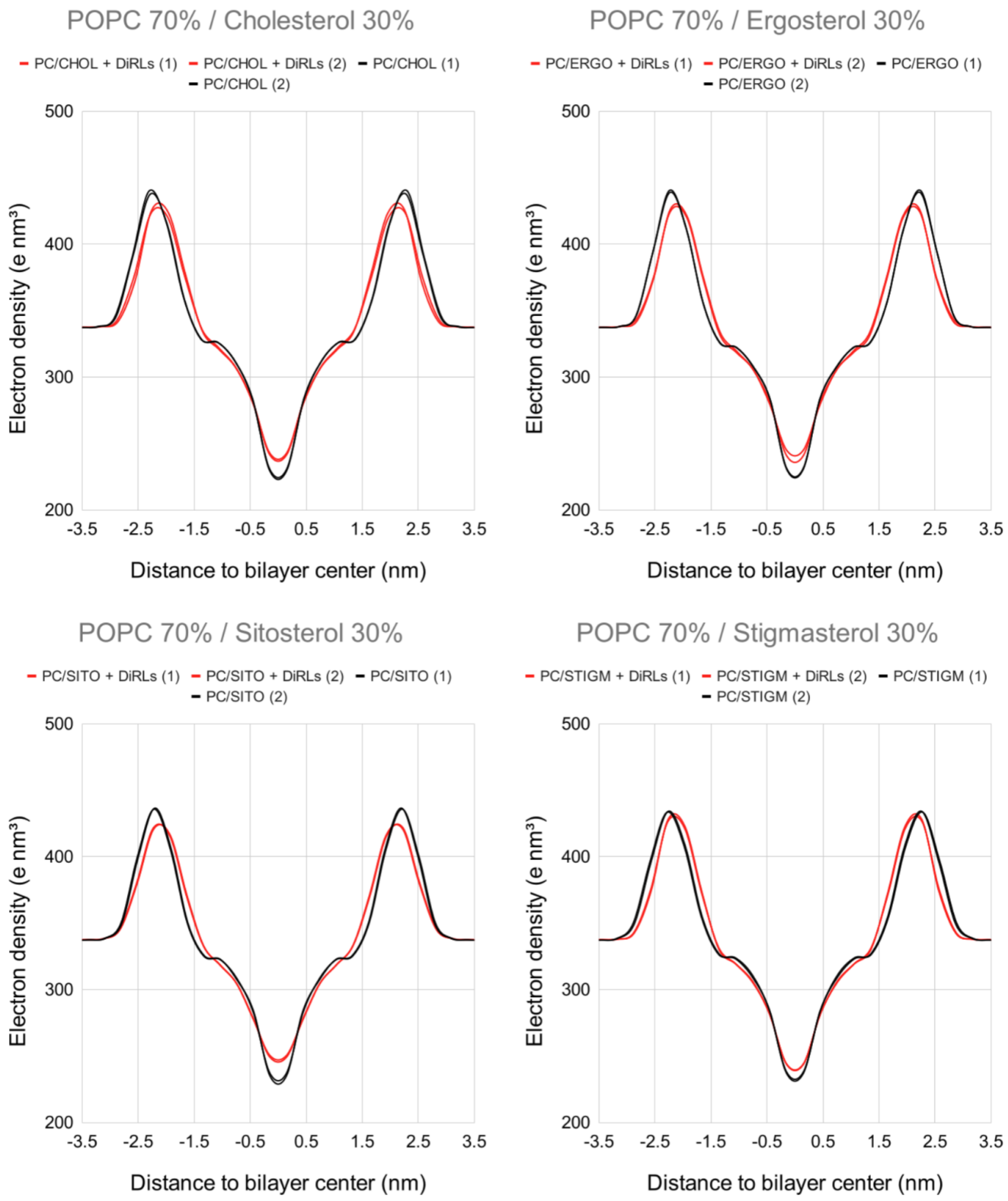

Figure S15. Electron density profiles for bilayers of various lipid compositions as calculated from MD simulations with and without RLs.

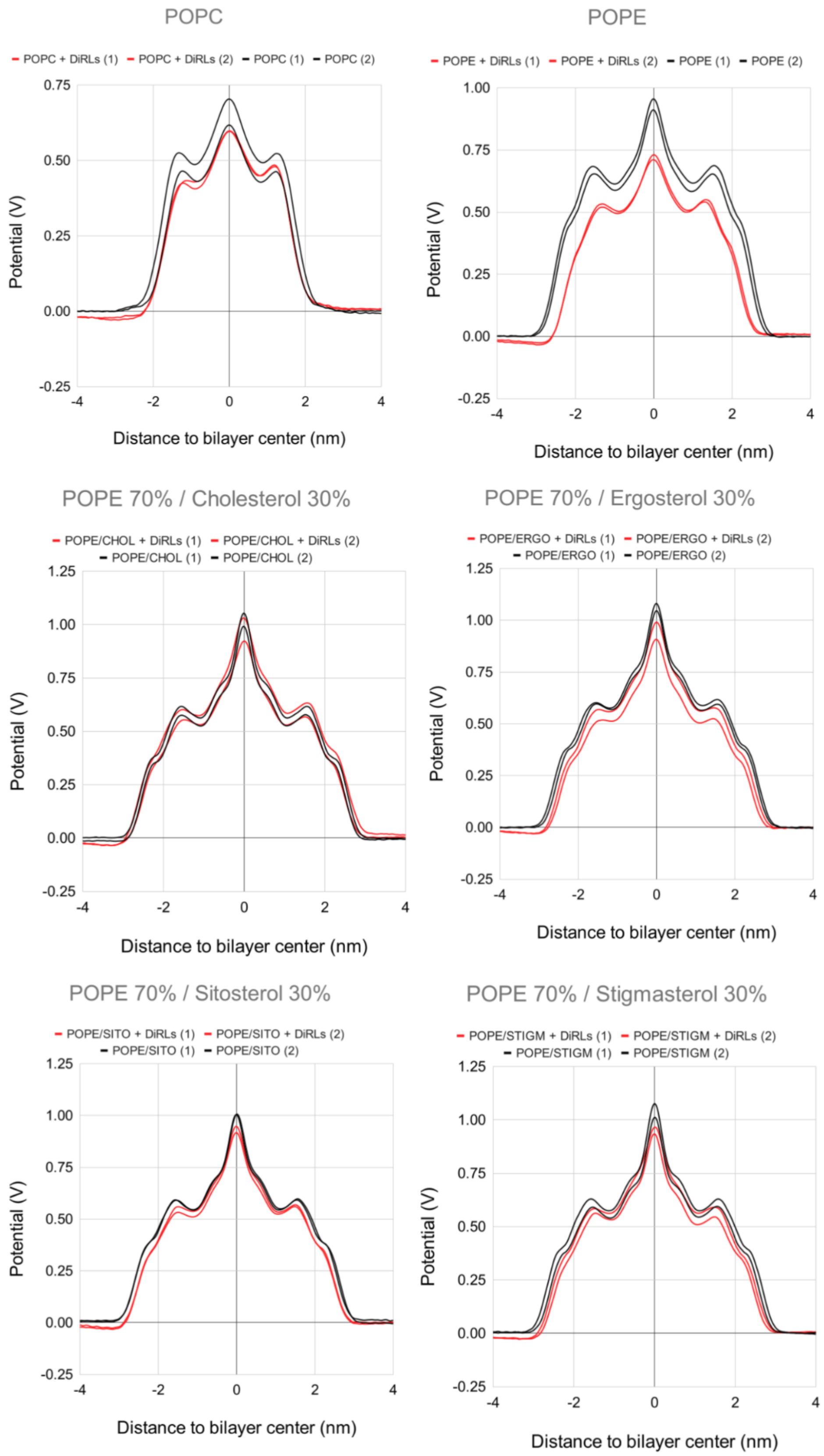

Figure S16. Dipole potential profiles for bilayers of various lipid compositions as calculated from MD simulations with and without RLs.

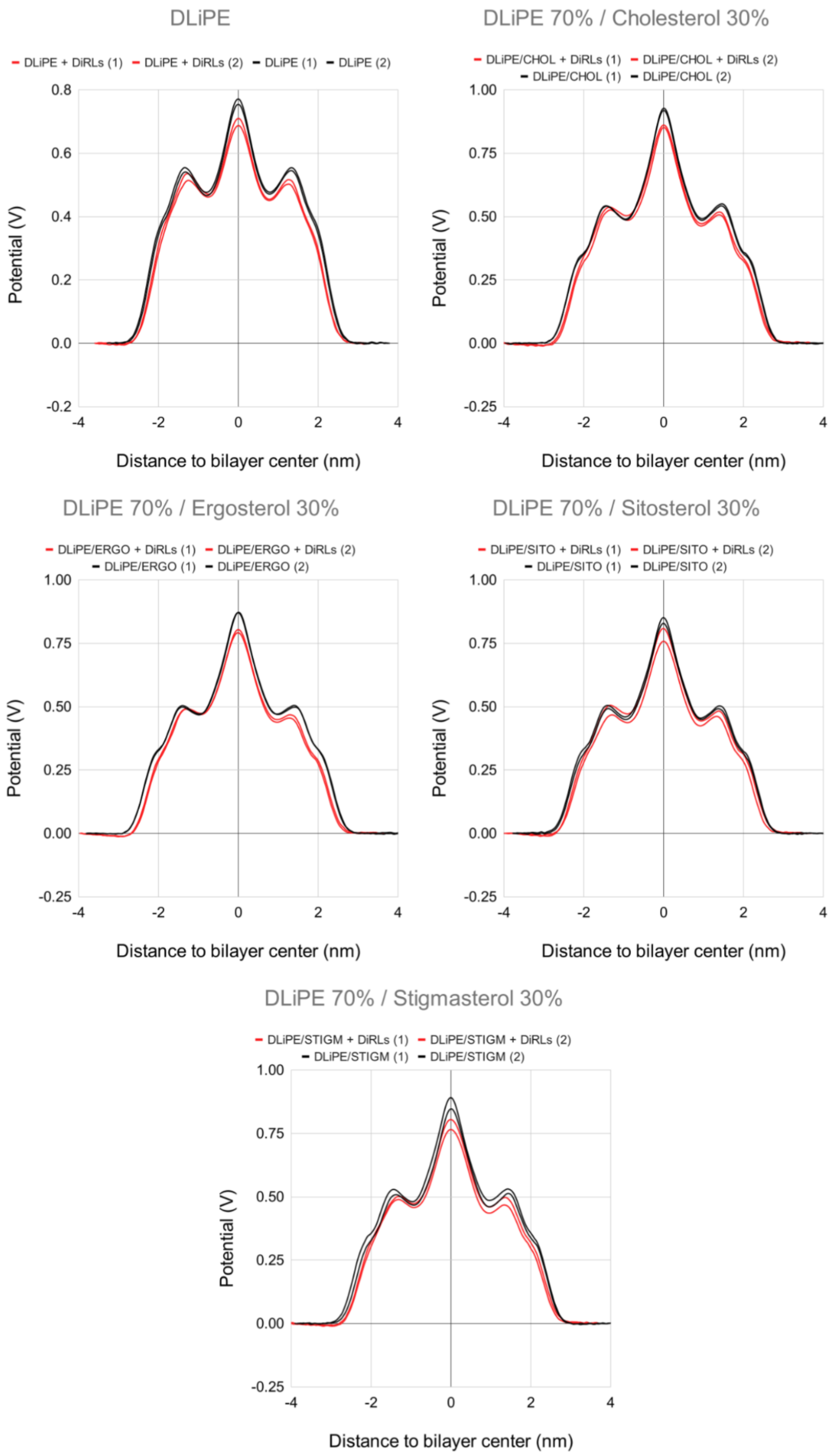

Figure S17. Dipole potential profiles for bilayers of various lipid compositions as calculated from MD simulations with and without RLs.

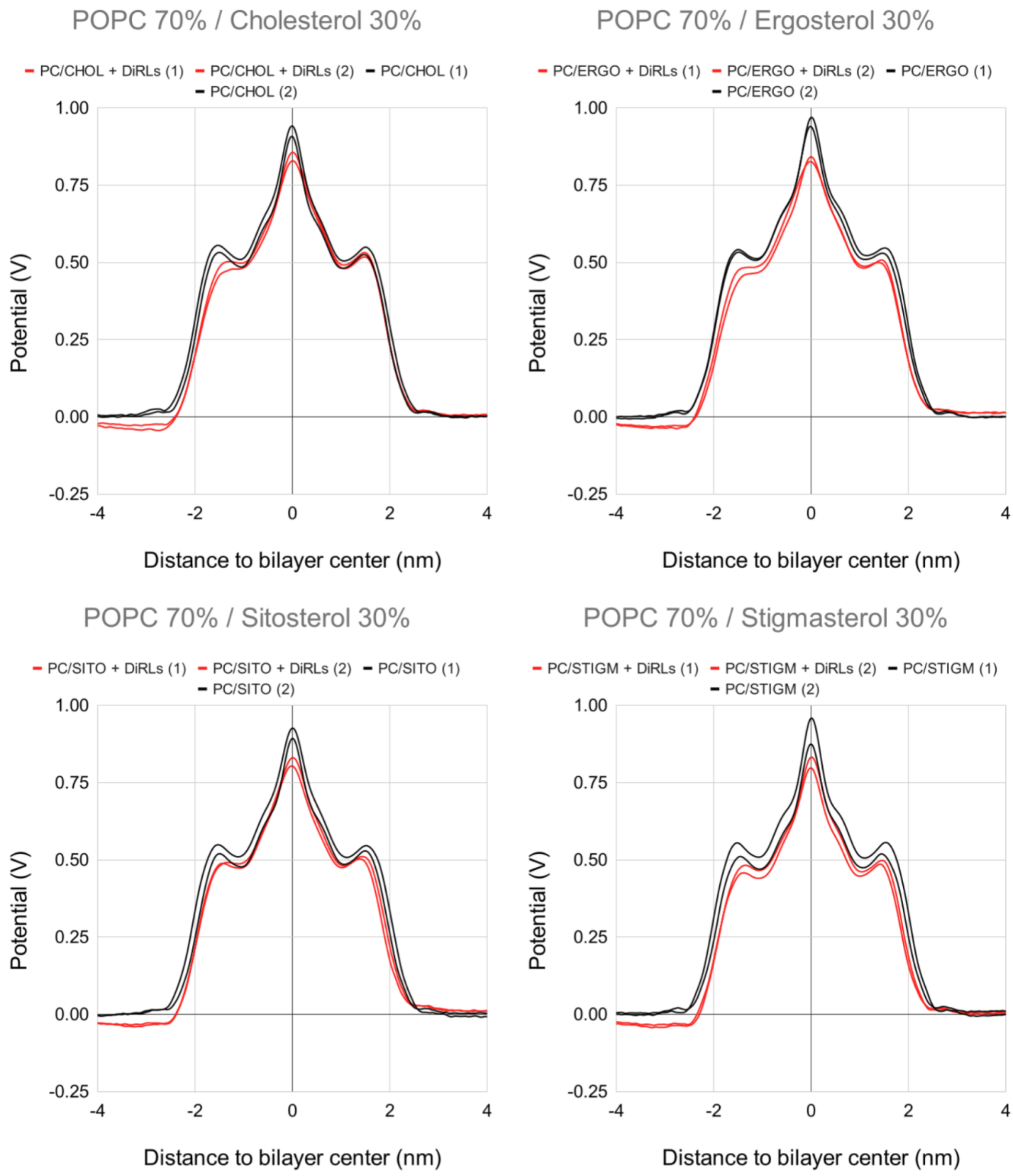

Figure S18. Dipole potential profiles for bilayers of various lipid compositions as calculated from MD simulations with and without RLs.

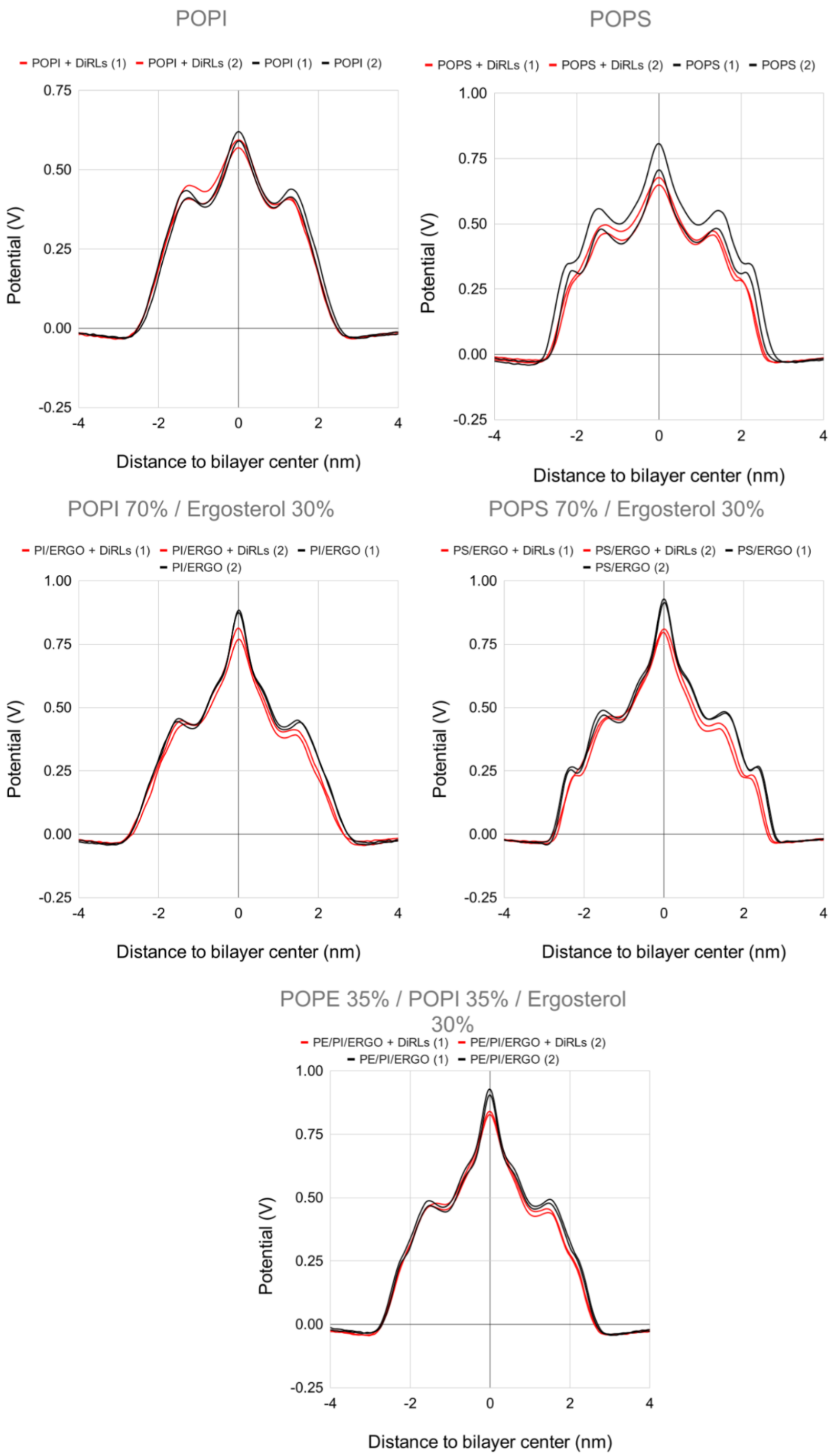

Figure S19. Dipole potential profiles for bilayers of various lipid compositions as calculated from MD simulations with and without RLs.

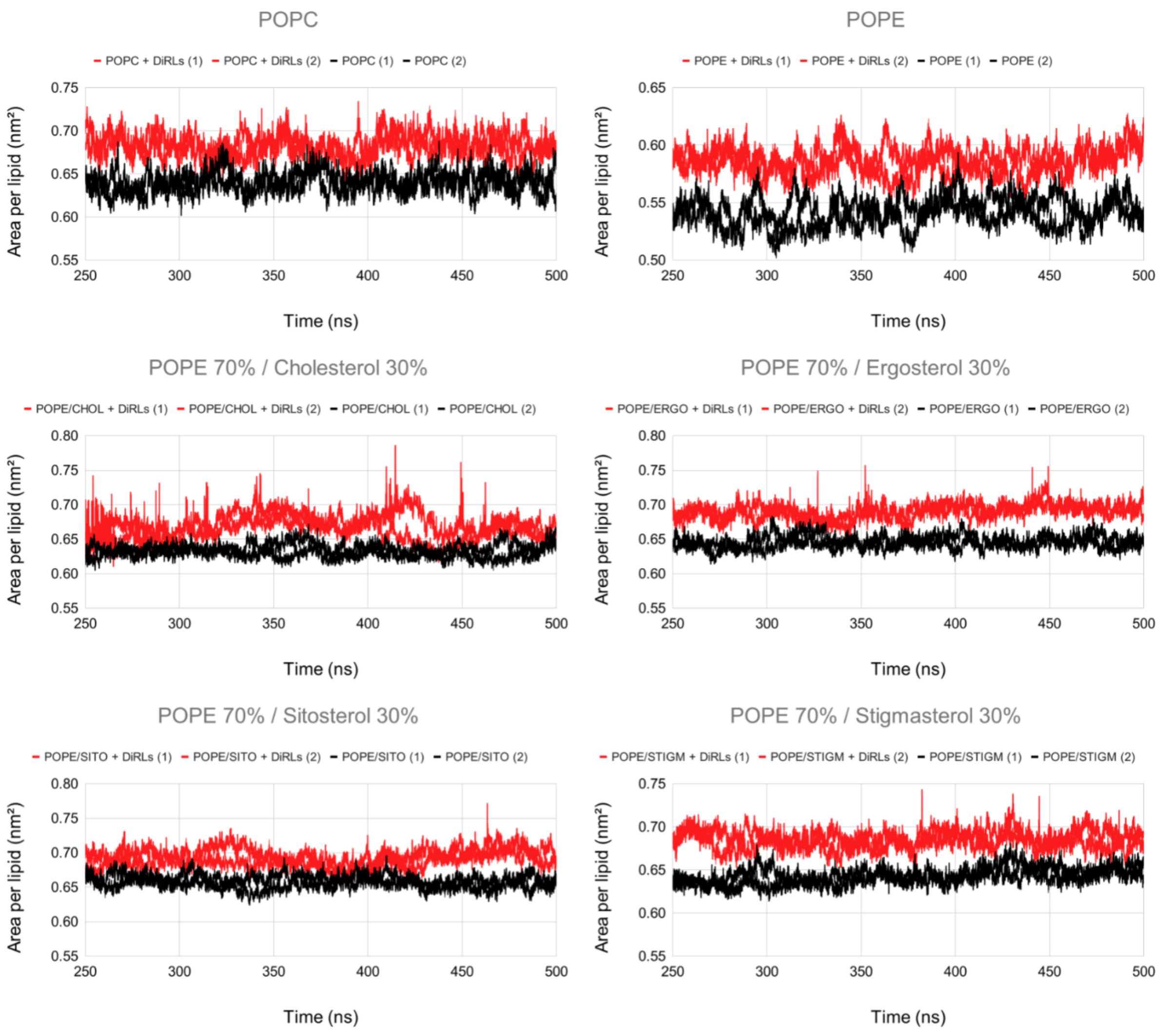

Figure S20. Area per lipid (nm^2) in bilayers containing various phospholipids compositions as calculated from MD simulations with and without RLs.

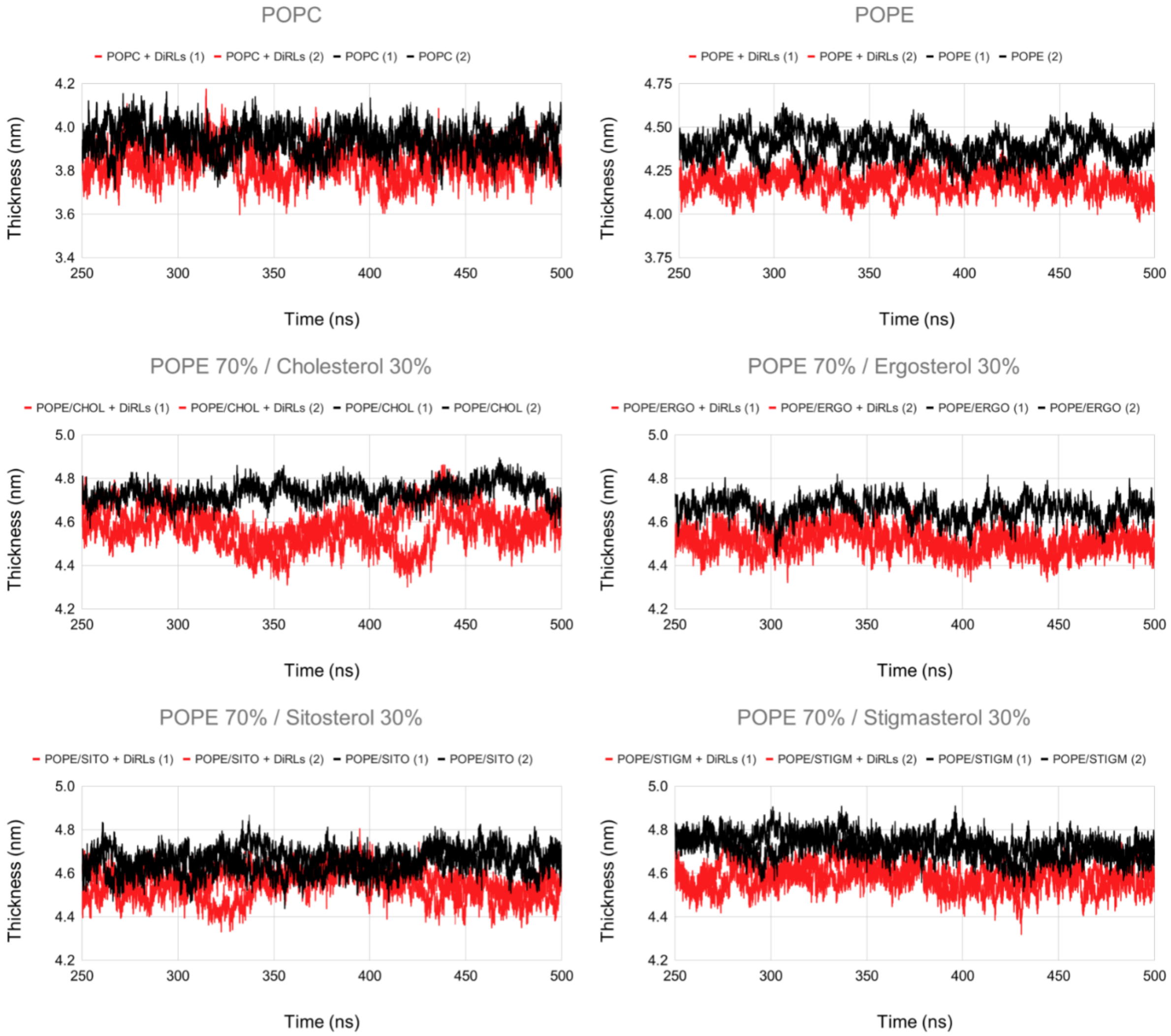

Figure S21. Thickness (nm) in bilayers containing various phospholipids compositions as calculated from MD simulations with and without RLs.
